# A mathematical model provides mechanistic links to temporal patterns in *Drosophila* daily activity

**DOI:** 10.1186/s12868-016-0248-9

**Published:** 2016-04-18

**Authors:** Andrey Lazopulo, Sheyum Syed

**Affiliations:** Department of Physics, University of Miami, 1320 Campo Sano Avenue, Coral Gables, FL 33146 USA

**Keywords:** Mathematical model, Power spectrum, Locomotion, *Drosophila melanogaster*, Circadian rhythms, Ultradian rhythms, Clock, Neuropeptide, Sleep

## Abstract

**Background:**

Circadian clocks are endogenous biochemical oscillators that control daily behavioral rhythms in all living organisms. In fruit fly, the circadian rhythms are typically studied using power spectra of multiday behavioral recordings. Despite decades of study, a quantitative understanding of the temporal shape of *Drosophila* locomotor rhythms is missing. Locomotor recordings have been used mostly to extract the period of the circadian clock, leaving these data-rich time series largely underutilized. The power spectra of *Drosophila* and mouse locomotion often show multiple peaks in addition to the expected at *T* ~ 24 h. Several theoretical and experimental studies have previously used these data to examine interactions between the circadian and other endogenous rhythms, in some cases, attributing peaks in the *T* < 24 h regime to ultradian oscillators. However, the analysis of fly locomotion was typically performed without considering the shape of time series, while the shape of the signal plays important role in its power spectrum. To account for locomotion patterns in circadian studies we construct a mathematical model of fly activity. Our model allows careful analysis of the temporal shape of behavioral recordings and can provide important information about biochemical mechanisms that control fly activity.

**Results:**

Here we propose a mathematical model with four exponential terms and a single period of oscillation that closely reproduces the shape of the locomotor data in both time and frequency domains. Using our model, we reexamine interactions between the circadian and other endogenous rhythms and show that the proposed single-period waveform is sufficient to explain the position and height of >88 % of spectral peaks in the locomotion of wild-type and circadian mutants of *Drosophila*. In the time domain, we find the timescales of the exponentials in our model to be ~1.5 h^−1^ on average.

**Conclusions:**

Our results indicate that multiple spectral peaks from fly locomotion are simply harmonics of the circadian period rather than independent ultradian oscillators as previously reported. From timescales of the exponentials we hypothesize that model rates reflect activity of the neuropeptides that likely transduce signals of the circadian clock and the sleep–wake homeostat to shape behavioral outputs.

**Electronic supplementary material:**

The online version of this article (doi:10.1186/s12868-016-0248-9) contains supplementary material, which is available to authorized users.

## Background

Biological oscillators with periods varying from seconds to years play important roles for most living organisms [1]. These oscillators have been divided into three groups by period length: (1) circadian oscillators, with periods close to 1 day; (2) ultradian oscillators, with periods less than 24 h; and (3) infradian oscillators, slower than circadian oscillators, with periods from a few days to a few seasons. Examples of ultradian rhythms can be found in the oscillation of body temperature in golden hamsters and in electroencephalogram measurements from the human brain [2, 3]. Infradian oscillations can be seen in animals, such as birds, that have annual migration cycles [4]. Behavioral oscillators form the group of oscillators that are responsible for adaptation to external environments, control of daily cycles of activity, annual reproductive rhythms, and migrations [5]. The most prominent and well-studied of the behavioral oscillators is the circadian clock [6–8].

The circadian clock is an endogenous biological oscillator with a period of approximately 24 h that controls daily activity and is found in most plants and animals. This clock helps to synchronize an organism’s internal processes and behavioral outputs with the daily light–dark cycle. The fruit fly *Drosophila melanogaster* is one of the primary model organisms in the study of behavioral rhythms and previous studies in this invertebrate have revealed the core components of the circadian clock that have been subsequently found to be conserved in other organisms, including mammals [9].

How these overt behavioral rhythms are generated has been an active topic of research for decades. One of the first theoretical models for the circadian system was proposed by Pittendrigh, who suggested that the circadian clock consists of multiple oscillatory components that maintain identical frequency and appropriate phasing through external entrainment [10]. An alternative mechanism was proposed by Pavlidis who relied on contemporary research showing that most known biochemical oscillators had periods which did not exceed a few minutes [11]. Pavlidis showed mathematically that a system of strongly coupled oscillators with short periods can produce a robust circadian rhythm and simulate many aspects of circadian behavior. The Pavlidis mechanism was supported subsequently by various mathematical models in which circadian rhythms result from a coupling of ultradian oscillators with periods of a few hours [12, 13]. In yet another theoretical study circadian rhythms were generated by coupling oscillators with periods of only a few seconds [14].

Behavioral studies of various animals claim to reveal multiple ultradian rhythms in daily activity [15–21]. These studies are commonly performed using spectral analysis of activity recordings. Power spectra typically show multiple peaks at periods of less than 24 h, which some studies have attributed to ultradian oscillators. In these behavioral studies short ultradian rhythms associated with foraging activity were observed in Siberian hamsters [15] and common voles [16]. The animals were studied under conditions disruptive to circadian rhythms, leading the authors to suggest that these ultradian rhythms are independent of the circadian clock. Other research has shown periods between 4 and 12 h in the activity of inbred strains of mice [17]; these rhythms were observed both in 12 h light/12 h dark conditions and in constant darkness. A more recent report from Blum et al. on dopamine-dependent oscillations in mouse activity with a ~4 h period has reinvigorated the discussion on ultradian rhythms in mammalian behavior [18]. In studies of fruit fly activity, ultradian rhythms were first claimed in *per*^0^ flies measured under constant conditions [19, 20]. Circadian rhythms are abolished in *per*^0^ animals due to a mutation in the *period* (*per*) gene [22]. Subsequently, ultradian rhythms were also reported in wild type and other clock mutants of *Drosophila melanogaster* [21]. Based on these data, the authors proposed that in fruit flies *per* couples ultradian oscillators and ultimately produces a circadian rhythm. However, even though it was not explicitly mentioned, power spectra in these studies were interpreted under the assumption that only one spectral peak results from the circadian clock and therefore the activity data have a sinusoidal shape. In reality, rhythmic behavioral data often have a non-sinusoidal shape in time.

The core of the *Drosophila* circadian clock consists of four proteins, PERIOD (PER), TIMELESS (TIM), CLOCK (CLK) and CYCLE (CYC), that form a negative feedback loop [7]. Although the molecular components differ, circadian clocks in mammals and plants also employ similar genetic architecture and, in each case, this conserved architecture produces clock gene oscillations that are sinusoidal in shape with period of approximately 24 h [8, 23–26]. These molecular oscillations converge on downstream behavioral circuits such as those for sleep, feeding and mating, and ultimately shape daily activity of organisms [3, 27, 28]. In *Drosophila*, temporal patterns in solitary activity are generally studied using locomotion measurements by detecting a fly crossing an infra-red light beam in the middle of a tube. A typical recording shows a non-sinusoidal time series with two distinguishable peaks: the morning peak (M), which starts during the end of the night time and has its maximum when the light turns on; and the evening peak (E), which starts during the end of the day and reaches its maximum when the light turns off. The M and E activity peaks are thought to be produced by two groups of pacemaker neurons, the M and E oscillator cells, and modulated by neuropeptides secreted by the pacemakers [29–32]. These two groups of neurons produce different neuropeptides. The neuropeptide produced in the morning oscillator cells is pigment dispersing factor (PDF) which has been shown to synchronize clock neurons and promote morning activity in fly behavior [33]. One of the neuropeptides produced by the evening oscillator cells is called the ion transport peptide (ITP) which is thought to influence fly activity around dusk [34]. A second possible source for the non-sinusoidal shape is the fly sleep homeostat, a feedback system that keeps track of sleep need in the animal. In mammals, the homeostat output has been modeled as repeating patterns of exponential rise and decay [35]. Assuming the fly sleep homeostat produces similar patterns, interaction of its output signal with that of the clock could shape the final behavioral output as a non-sinusoidal waveform, as seen in locomotor recordings. Indeed, that sleep–wake signals influence locomotor output is a standard assumption in fly studies which now routinely use locomotion as a read-out of sleep and wakefulness [36]. These possibilities suggest that understanding the temporal shape of the recordings could provide systems-level access to the biochemical pathways that influence fly locomotion patterns.

A mathematical description of the widely studied daily locomotion patterns in flies is currently missing and its absence has limited our interpretation of the data in both frequency and time domains. In frequency space, spectral data of locomotion are typically interpreted without considering the shape of the activity time series, although it is well-known from Fourier’s theorem that the form of the time series is a critical determinant of its power spectrum. For example, non-sinusoidal signals with a single periodicity, such as square or triangular waves, produce power spectra with multiple peaks, which correspond to the harmonics of the primary period. In the time domain, beyond a qualitative description of how M and E peak heights vary with simple perturbations, no biophysical description exists for the peculiar shape of fly locomotor activity. For instance, we do not know what biological processes control the rates of increase and decrease of activity around M and E peaks.

We address these limitations by constructing a simple waveform with a single period that closely resembles the shape of fruit fly activity. Our model consists of four exponential terms that generate a pattern resembling the M and E peaks. The power spectrum of the proposed waveform has peaks at harmonics of the primary period $T_{0}$ and we demonstrate that the predicted spectral peaks can account for the multiple periodicities seen in the power spectrum of *Drosophila* locomotion. The data show that lesions in circadian genes that shift the circadian peak also shift the ultradian peaks, in accordance with our model. Genetic or environmental ablation of the circadian clock expectedly eliminates the circadian spectral peak but also reduces to noise levels the height of peaks in the ultradian range. Thus we weaken the competing view that circadian rhythms may arise from the coupling of ultradian oscillators by showing that the secondary spectral peaks in *Drosophila* locomotion, rather than being produced by independent oscillators, simply result from the non-sinusoidal shape of fruit fly activity. In the time domain, we determine the model exponential rate constants for wild-type and clock mutants of *Drosophila*. These rates which vary widely in magnitude between 0.024 and 14.5 h^−1^, with mean value of 1.49 h^−1^, are found to be independent of the pace of the circadian clock. Guided by these results, we propose that the exponents might come from the accumulation and release rates of neuropeptides important in regulating activity and sleep in the fly brain. Finally, we use the model to make quantitative predictions about candidate biological processes in general that could give rise to the rate constants and suggest future experiments to help identify the associated molecular substrates.

## Results

**Fig. 1:**
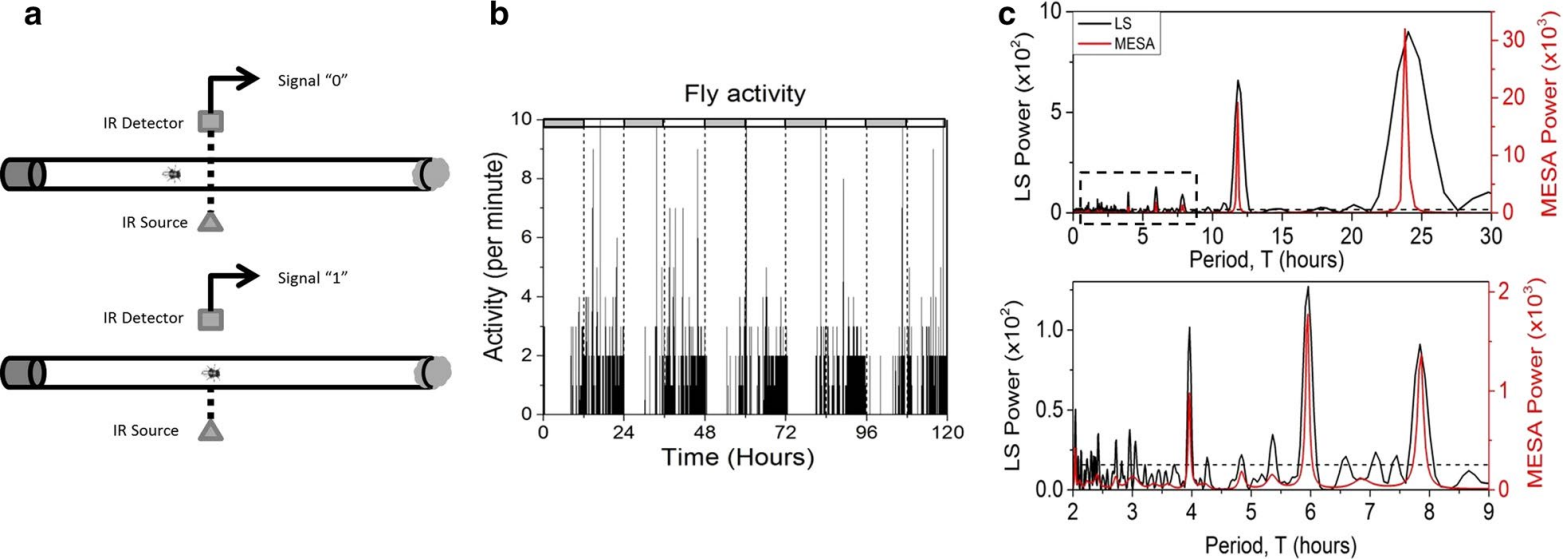
Power spectrum of fly locomotor activity shows multiple peaks. **a** Schematic representation of fly activity measurement. Each time the enclosed fly crosses an IR beam, the computer receives a “1”. **b** Activity of a single wild type fruit fly measured in constant darkness. Subjective day/night time is shown in *white*/*grey bars*. **c** Power spectrum of the fly activity. Spectrum from 0 to 30 h is shown on *top*, *dashed rectangle* is enlarged in the *lower panel*. Power spectra calculated with Lomb–Scargle (LS, *black*) and maximum entropy spectral analysis (MESA, *red*) methods show the expected ~24 h circadian peak and additionally show a series of statistically significant peaks at lower $T$. *Dashed horizontal line* represents statistical significance of 0.005 for the Lomb–Scarge spectrum. Peak positions detected by LS and MESA agree to within 2 %

Fruit fly activity was measured using the standard *Drosophila* Activity Monitor (DAM). Each fly is placed in an individual tube with food on one end and cotton on the other (Fig. 1a). An infrared beam crosses the tube in the middle in a perpendicular direction. When a fly interrupts the beam, the monitor receives a signal which is accumulated over time and sent to the computer every 20 s (Fig. 1b). Power spectra for activity data were calculated using maximum entropy spectral analysis (MESA) and Lomb–Scargle periodogram (LS) (Fig. 1c). Spectra obtained by each method show the expected 24 h peak. Additionally, there is a series of statistically significant peaks at smaller periods ($T$) with peak values from the two methods agreeing with each other to within 2 %. Unlike MESA, the Lomb–Scargle periodogram has an easily computable significance metric, which allows one to distinguish between significant and insignificant periodicities in the power spectra. Departing from the practice of filtering data prior to spectral analysis, we determine all power spectra directly from raw data. A number of past studies on ultradian rhythms adopted digital filtering as a standard procedure in their analyses [18, 21, 37–39]. However, as we demonstrate in this work (see “Methods”; Additional file 1: Fig. S9 and associated text), application of digital filters can irrevocably modify statistical properties of a time series and can give rise to artifacts in its power spectrum.

**Fig. 2:**
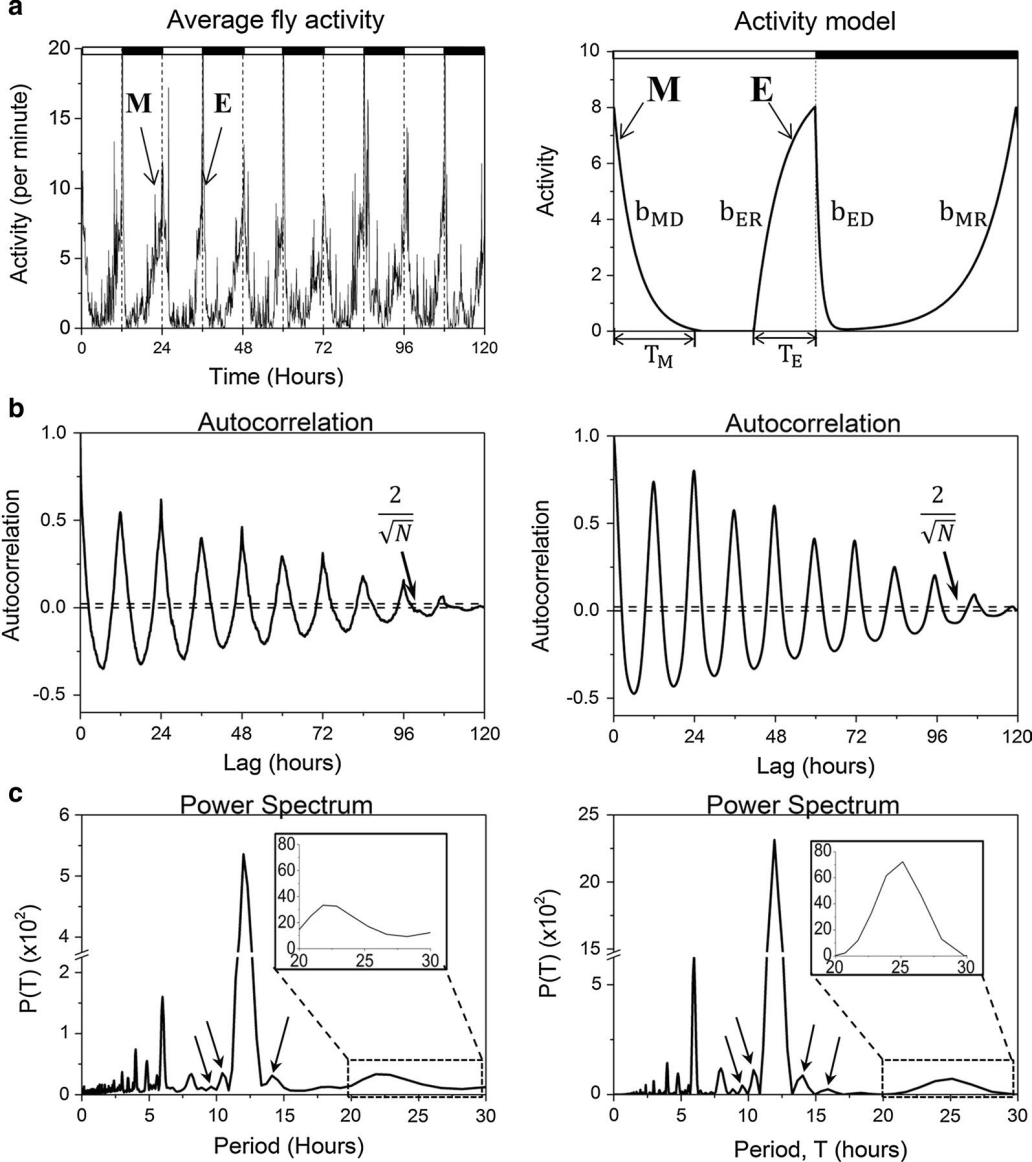
Comparison of data (*left*) to the model (*right*). **a** Average recording of 14 wild type flies measured in LD for 5 days; day/night shown with *white*/*black bars*. Model consists of four exponential terms with rates $b_{MD} ,\,b_{MR}$, $b_{ER}$, $b_{ED}$, and widths of morning and evening peaks given by $T_{M}$ and $T_{E}$. **b** Autocorrelations of data and model with primary period 24 h. Periods in signal are found from regularly appearing peaks with high correlation. Peaks exceeding the *dashed line* $2/\sqrt N$, where *N* is number of data points in activity trace, represent strong correlations. Strong 24 and 12 h periods are seen in both graphs. **c** Lomb–Scargle power spectra for the data and the model. Model reproduces strong peaks as well as small side peaks (*arrows*), which are from the Dirichlet kernel (see Additional file 1). Parameters used for this simulation: $b_{MD} = - 0.81\,{\text{h}}^{ - 1} ,\,b_{MR} = 0.486\,{\text{h}}^{ - 1} ,\,b_{ER} = 0.09 \,{\text{h}}^{ - 1} ,\,b_{ED} = 3.6\,{\text{h}}^{ - 1} ,\,T_{M} = 5.3\,{\text{h}},\,T_{E} = 3.6\,{\text{h}}.$

Figure 2a shows population averaged fly activity obtained from measurement of 14 flies in simulated light–dark conditions (LD) for 5 days. *Drosophila* daily activity has two distinguishable peaks. The morning peak (M) starts during the night and has a maximum when the light turns on and the evening peak (E) starts during the day and has a maximum when the light turns off. We constructed a model with a single fundamental period which reproduces these features of the activity. Our model consists of four normalized exponential terms with rates $b_{MD} ,b_{MR} ,b_{ED} , \,{\text{and}} \,b_{ER}$, with subscripts denoting morning decay (MD), morning rise (MR), evening decay (ED) and evening rise (ER):

$$F(t) = \left\{ \begin{array}{*{20}ll} \frac{{e^{{b_{\small MD} T_{\small M} }} - e^{{b_{\small MD} t}} }}{{e^{{b_{\small MD} T_{\small M} }} - 1}} , &\quad 0 < t < T_{\small M} \\ \frac{{e^{{b_{\small MR} \left( {t - T_{\small M} } \right)}} - 1}}{{e^{{b_{\small MR} \left( {T_{\small 0} - T_{\small M} } \right)}} }} , &\quad T_{\small M} < t < T_{\small 0} \\ \frac{{1 - e^{{ - b_{\small ER} \left( {t - \frac{{T_{0} }}{2} - T_{\small E} } \right)}} }}{{1 - e^{{ - b_{\small ER} T_{\small E} }} }}, &\quad \frac{{T_{\small 0} }}{2} - T_{\small E} < t < \frac{{T_{\small 0} }}{2} \\ e^{{ - b_{\small ED} \left( {t - \frac{{T_{\small 0} }}{2}} \right)}} , &\quad \frac{{T_{\small 0} }}{2} < t < T_{\small 0} \end{array} \right.$$

In the proposed waveform, exponential terms with rates $b_{MD}$ and $b_{MR}$ form the M peak with width $T_{M}$ while terms with rates $b_{ER}$ and $b_{ED}$ form the E peak with width $T_{E}$ (Fig. 2a right). Together, these four exponents create a wave with $T_{0} = 24\,{\text{h}}$. The Fourier transform of $F(t)$ contains terms proportional to ${\text{cos}}\left( {2\pi \frac{{T_{0} }}{T}} \right)$ and ${\text{sin}}\left( {2\pi \frac{{T_{0} }}{T}} \right)$, which produce peaks in the power spectra at harmonics of the primary period $T_{0}$ (see Additional file 1). To estimate peak heights, we analytically calculated Fourier transform only at harmonics $T_{n} = T_{0} /n$ of the primary period. The analytic expression has multiple terms such as those containing $\cos \left( {\frac{{2T_{M} n\pi }}{{T_{0} }}} \right)$, $\sin \left( {\frac{{2T_{E} n\pi }}{{T_{0} }}} \right)$, $e^{{T_{M} b_{MD} }}$, and $e^{{T_{E} b_{ER} }}$ (see Additional file 1: equations 5–7).

Since a mathematical function that adequately reproduces fly locomotor data has been lacking, we tested different waveforms to simulate locomotion and interpret its power spectrum. In previous work, it was assumed that behavioral rhythms have the form of a square wave [37]. Although a square wave with period $T_{0}$ produces multiple spectral peaks (Additional file 1: Fig. S1), it is able to interpret only odd-numbered harmonics in spectral data and does not completely mimic fly activity. We also tested a sawtooth wave, which is able to interpret most spectral peaks, but does not describe with high fidelity the shape of *Drosophila* locomotion data. Fruit fly circadian behavior is controlled by the activity of clock neurons, which often have exponential patterns of activation and deactivation [40]. The underlying exponential kinetics and the observed shape of activity data motivated us to build a model that consists of exponential terms.

In order to find similarities between the model and data and to look for the main periodicities in data, we first calculated the autocorrelation function. Autocorrelation with 1 min time lags was obtained by calculating the covariance of each time series with itself. The periods of the time series can be found from regularly appearing peaks of high correlation. Even though both the model and the data have a primary period of 24 h, the autocorrelations show strong 24 and 12 h periodicities (Fig. 2b). The 12 h periodicity results from the 12 h time interval between M and E peaks. Having obtained a basic level of similarity between data and model, we next analyzed them with the Lomb–Scargle periodogram to calculate a high-resolution power spectrum (Fig. 2c). Similar to the actual fly recordings, the proposed waveform with the primary period of 24 h shows multiple peaks in the power spectrum at values between 0 and 24 h with the 12 h peak dominating. The 12 h periodicity is significantly increased by the externally imposed 12-h light/12-h dark cycle. The model power spectrum not only reproduces prominent peaks from the data, but also the smaller peaks that appear near the prominent peaks. These small peaks, whose number and position can be predicted from the Dirichlet kernel, arise from the fact that we work with finite discrete data (Additional file 1: Fig. S8). For both the experiment and the simulation, the circadian peak is ~24 h.

**Fig. 3:**
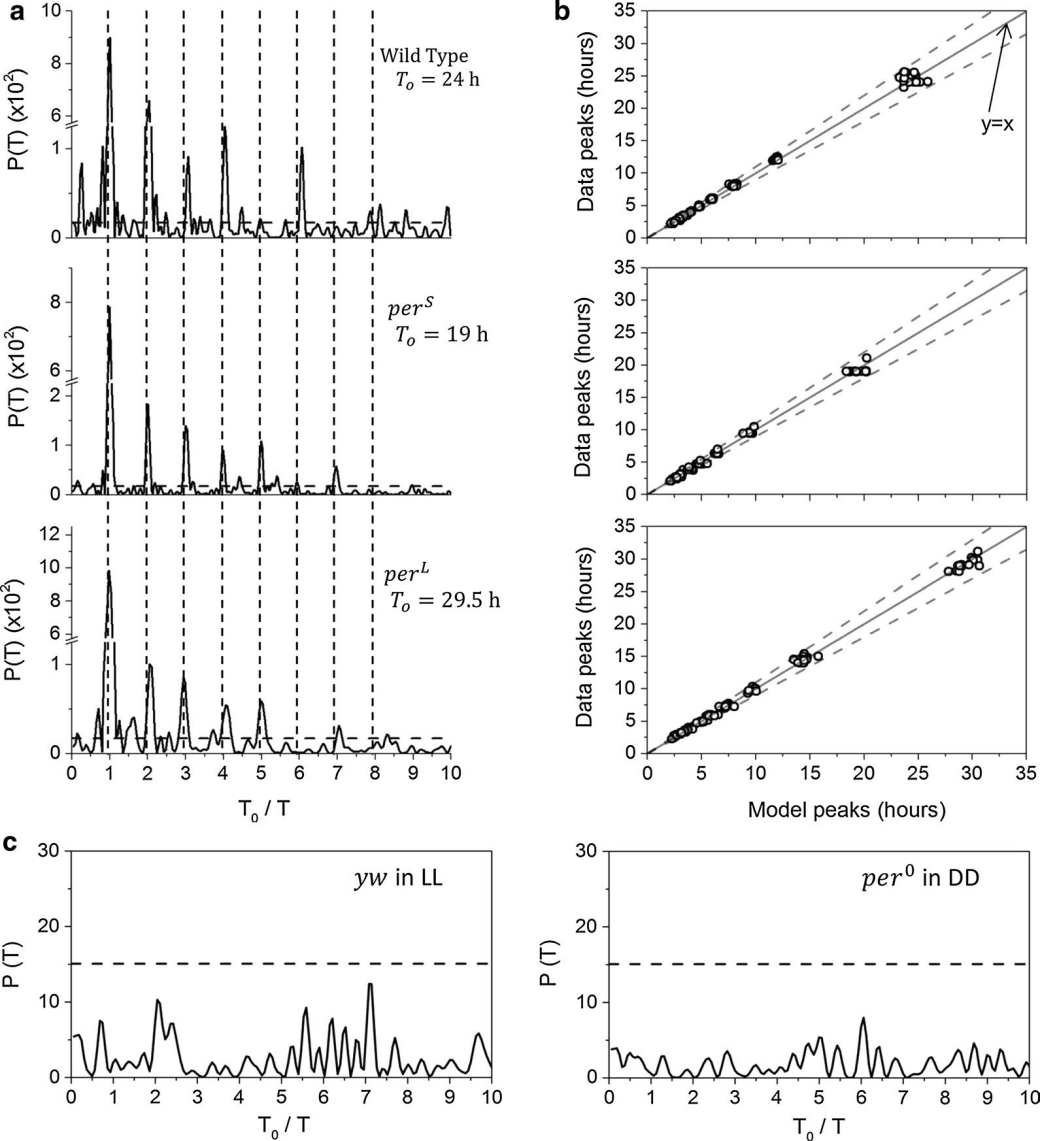
Our model correctly predicts majority of peaks in power spectrum of fly locomotion. **a** Power spectra of individual wild type and clock mutants of *Drosophila* measured in constant darkness for 5–7 days. *X*-axis given as ratio $T_{0} /T$, with the circadian period $T_{0}$ indicated in each case. Increasing values indicate shorter periods of oscillation. For each $T_{0}$, prominent secondary peaks are found at $T_{0} /T = 2,3, \ldots$ accompanied by lower power Dirichlet kernel peaks. **b** Comparison of peaks detected in the data to peaks predicted by the model was obtained by analyzing wt (N = 29), *per* ^*S*^ (N = 22) and *per* ^*L*^ (N = 19) flies. Only peaks higher than $p = 0.005$ were used in the analysis. $y = x$ is shown as a *solid line*, 10 % deviation shown as *dashed lines*. For wild type and clock mutants more than 88 % of the data peaks for *T* = 2–35 h can be explained by the model with ± 10 % error. **c** Power spectra of a wild type fly measured in LL and a *per* ^*0*^ mutant measured in DD. Neither spectrum shows peaks higher than $p = 0.005$ significance level (*dashed line*). For both graphs $T_{0} = 24$  h was used for scaling the abscissa

We noticed that the majority of peaks that appear in the power spectra of wild-type flies measured in LD occur at multiples of the primary period $T_{0}$. To test if the secondary peaks result from the externally imposed light/dark cycle, we measured fly (N = 29) locomotion in constant darkness (DD) for 5–7 days. This analysis revealed that together with the 24 h peak reflecting the circadian clock, the power spectrum still has the same additional peaks that appear at multiples of $T_{0}$ (Fig. 3a, top), suggesting that the additional periodicities in the power spectra of fly activity are simply harmonics of the endogenous circadian period $T_{0}$.

To further test our assumption that multiple spectral peaks of fly locomotion all result from the circadian clock, we used different circadian period mutants of *Drosophila melanogaster*. We analyzed *per*^*S*^ (N = 22) and *per*^*L*^ (N = 19) flies, with average circadian periods of 19 and 29.5 h (Additional file 1: Fig. S10), respectively, for the presence of secondary peaks in their power spectra. Activity of both genotypes was measured in constant darkness for 7 days and analyzed with Lomb–Scargle periodogram. Power spectra of the clock mutants show secondary peaks that, similar to wild-type, were found to be at multiples of the mutant $T_{0}$ (Fig. 3a, middle and bottom panels). Almost all peaks in the power spectra of the different genotypes line up after rescaling the period axis with the circadian period. These results predict that elimination of the clock should abolish all rhythmicity in the power spectrum. We tested this prediction both by measuring circadian null mutants and by rendering wild-type flies arrhythmic by placing them in constant light (LL). Power spectra of *per*^0^ (N = 38) and *clk*^*Jrk*^ (N = 23) clock mutants that do not show circadian rhythmicity in constant darkness were determined from locomotor recordings of individual animals (Fig. 3c; Additional file 1: Figs. S2, S4) [22, 41]. The majority of *per*^0^ flies (N = 28) do not show statistically significant peaks at the *p* = 0.05 level between 2 and 35 h in the power spectra. Most *clk*^*Jrk*^ flies (N = 17) also do not have circadian rhythms and almost all peaks seen in the power spectra of wild-type flies between 2 and 35 h are absent in the power spectra of these mutant flies (Additional file 1: Fig. S2). We also measured activity of *yw* (N = 16) flies in LL and analyzed their power spectra (Fig. 3c; Additional file 1: Fig. S5). Most flies (N = 13) appear arrhythmic, showing no significant periodicities between 2 and 35 h. These results together provide strong support for the majority of spectral peaks resulting from the circadian clock producing a non-sinusoidal oscillation in fly behavior.

To test our model’s reliability, we compared peak positions in the power spectra for wild-type and shifted-period clock mutants of *Drosophila* to peak positions in the power spectra of model with different primary periods (Fig. 3b). For each genotype we constructed a model with $T_{0}$ matching the circadian period in the data, yielding harmonics at values $\frac{{T_{0} }}{1},\frac{{T_{0} }}{2}, \ldots ,\frac{{T_{0} }}{n}$ in the power spectra, which we then used to interpret peaks in the data. We used only peaks higher than the significance level of 0.005 in order to exclude Dirichlet kernel peaks from the analysis. For all three genotypes our simple model is able to identify more than 88 % of peaks in the data to within 10 % error (see “Methods” for details).

**Fig. 4:**
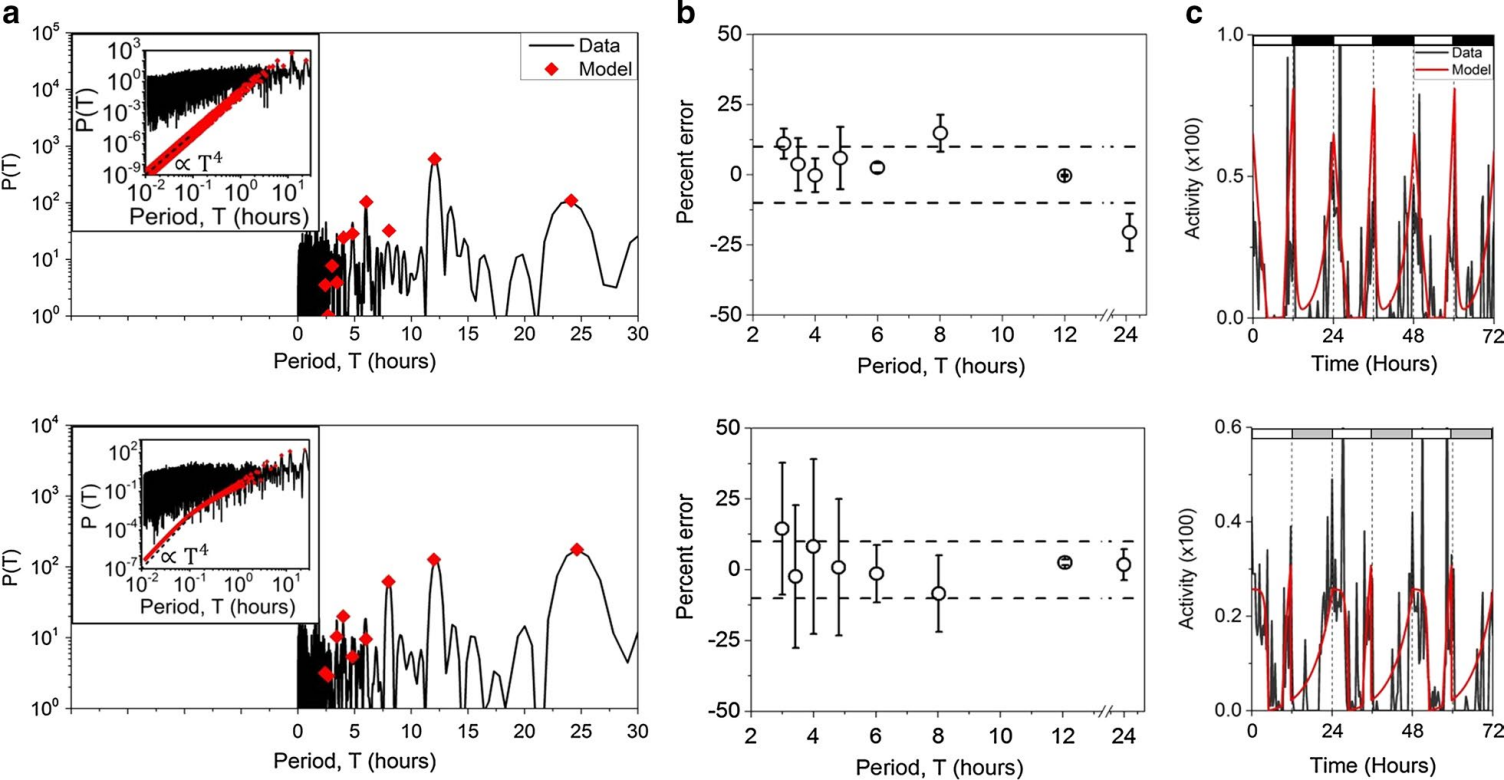
Model parameters are obtained by fitting the power spectrum. **a** Examples of data power spectrum (*black line*) and fit (*red diamonds*) for a wild type fly measured in LD (*top*) and DD (*bottom*). In the LD data, the 12 h peak is stronger than the 24 h peak and was therefore used as the reference peak in the fit. Comparison of model and data power spectra for low $T$ shown in the *inset*. For low values of the period, the model predicts peak height $H(T) \propto T^{4}$ (*inset*, *dashed line*), a behavior not shown by the data. **b** Errors in peak height estimation from 10 flies (mean error ± standard deviation). On average, peak heights are estimated with error of 10 % (*dashed lines*) or better. **c** Parameters obtained from **a** are used to construct model of fly activity. Data (*black line*) shown in 20 min bins; day/night shown with *white*/*black bars* for LD and subjective day/night shown with *white*/*grey bars* for DD

We next determined the exponents $b_{MD}$ − $b_{ED}$ for wild-type flies in DD and LD. In our model, these exponents define the shape of the M and E peaks. The model parameters were obtained from the power spectra of activity data (Fig. 4a). Spectra were fitted with an analytical expression $H(T_{n} )$ obtained by calculating the square of the Fourier transform of $F(t)$. The square of the Fourier transform yields peak heights $H(T_{n} )$ at harmonics $T_{n}$ of the primary period $(T_{n} = T_{0} /n)$ [see Additional file 1: equations (5)–(8)]. In DD data, $T_{0}$ was determined from the peak at the circadian frequency. Since the fitting procedure is sensitive to the initial choice of parameters, as an initial guess we used parameters from a preliminary fitting of activity data with the model. The exponents obtained from the fits shown in Fig. 4 are $b_{MD} = - 0.09\,{\text{h}}^{ - 1}$, $b_{MR} = 0.34 \,{\text{h}}^{ - 1}$, $b_{ER} = - 0.05\,{\text{h}}^{ - 1}$, and $b_{ED} = 1.83\,{\text{h}}^{ - 1}$ for LD, and $b_{MD} = 1.4 \,{\text{h}}^{ - 1}$, $b_{MR} = 0.24\,{\text{h}}^{ - 1}$, $b_{ER} = 0.50\,{\text{h}}^{ - 1}$, and $b_{ED} = 6.2\,{\text{h}}^{ - 1}$ for DD. The analytical expression also produced good fits for the activity power spectra with the average peak height fit error of less than 10 % (Fig. 4b) (see “Methods” for details). Final values of the parameters determined from fitting the power spectrum were then used to construct a model for the activity recordings (Fig. 4c). For the measured wild-type flies, the constructed model shows good fit for locomotor activity with the average rate constant magnitude ~1.2 ± 2 h^−1^ (mean ± standard deviation). The parameters $b_{MD}$ and $b_{ER}$ obtained from fitting data can be either positive or negative, which means that the exponential terms with $b_{MD}$ and $b_{ER}$ in $F(t)$ can be either concave or convex. Interestingly, we find that the parameters $b_{MR}$ and $b_{ED}$ are always greater than 0 and therefore corresponding terms are always concave. It should be noted here that our model does not impose any restrictions on the numerical values of the parameters.

While the predictive power of the model is reliable for $T > 2 \,{\text{h}}$, for $T < 2 \,{\text{h}}$ our model strongly deviates from data (Fig. 4a, inset). The calculated model peak heights $H(T_{n} )$ predict that for period values less than $2\pi /b$, where $b$ is the largest exponent, typically $b_{\text{MD}}$ or $b_{\text{ER}}$, the peak heights are proportional to $T^{4}$ (see Additional file 1). However, the data show weak sensitivity to *T* in this regime. There are a few factors that can affect the power spectrum of fruit fly activity at low period values. The first factor is that the locomotion measurement reports fly movement only when it crosses the middle of the tube. This lack of spatial resolution causes the system to miss small-scale movements which happen without the fly crossing the beam. A second factor is the Nyquist frequency. In any measurement the Nyquist frequency is equal to half of the sampling frequency and imposes a lower limit on the periods one can search for in a given time series. Thus, limitations imposed by both the measurement method and by the Nyquist frequency likely result in detectable discrepancies between the data and the model for low $T$. For these reasons, all our model-based predictions are restricted to *T* > 2 h.

**Fig. 5:**
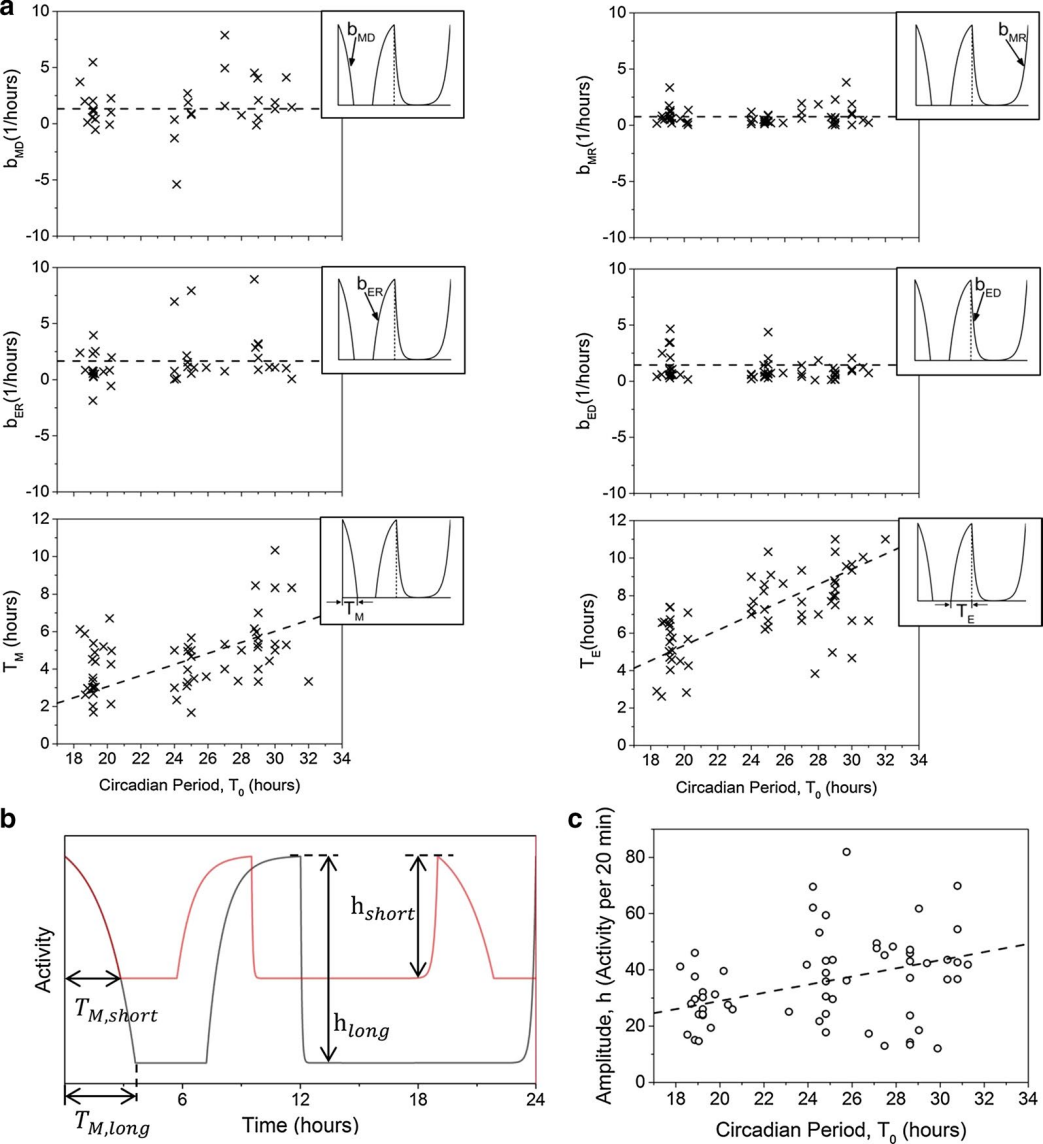
Analysis reveals quantitative relations between model parameters and circadian period. **a** Parameters extracted from fitting locomotor data of wild type (N = 11), *per* ^*S*^ (N = 22), *per* ^*L*^ (N = 19) and *tim* ^*UL*^ (N = 11) flies measured in constant darkness for 5–7 days. *Plotted parameter* is indicated in the adjacent *box* for *each graph*, with curvatures shown for $b_{MD} ,b_{MR} ,b_{ER} ,b_{ED} > 0$. The data show that the exponential rate constants are independent of $T_{0}$, while parameters $T_{M}$ and $T_{E}$ grow roughly linearly with $T_{0}$. *Dashed lines* are visual guides (*top* and *middle panels*) or linear fits (*bottom panels*). **b** Sketch of two locomotor patterns where the red locomotion is driven by a faster clock (shorter $T_{0}$). If increase in $T_{0}$ results in lengthening of activity peak widths from $T_{M,short}$ to $T_{M,long}$ without altering the exponential rates, our model predicts that the activity amplitude must also increase from $h_{short}$ to $h_{long}$. The first M peaks are shown to overlap to emphasize constancy of the exponential rates. The *red sketched* activity has been vertically shifted for visual clarity. **c** Data from flies in **a** demonstrate a positive correlation between average activity amplitude h and the circadian period $T_{0}$. *Dashed line* is a linear fit to the data giving ${\text{h}} \approx 1.4T_{0}$

In order to understand what factors affect the model parameters, we determined their values for different clock mutants and tested for their relation to the circadian period $T_{0}$. Wild-type, *per*^*S*^, and *per*^*L*^ animals introduced above together with *tim*^*UL*^ flies (N = 11) with average T_0_ ~ 27 h were used in these analyses (Additional file 1: Fig. S10). Given our mathematical description of fly activity, we hypothesized two possibilities: one, in which the rate constants do not vary but $T_{M}$ and $T_{E}$ adjust with $T_{0}$ and another, in which both sets of parameters change with $T_{0}$. Interestingly, the data show that the rate constants $b_{MD}$, $b_{MR}$, $b_{ER}$, $b_{ED}$ do not depend strongly on $T_{0}$ (${\text{Adj }}R^{2} < 0.1$ for linear fits), whereas the parameters $T_{M}$ and $T_{E}$ that determine width of the morning and evening peaks, increase with $T_{0}$ (Fig. 5a). Assuming the measured parameters represent characteristics of underlying biological processes, the robustness of the rate constants suggests that the pace of the clock likely does not alter the kinetics of these processes. On the contrary, tight association between $T_{M}$ and $T_{E}$ and clock speed suggests that the clock may regulate when these processes are initiated and terminated. The rate constants typically range between ±5 h^−1^ for $b_{MD}$ and $b_{ER}$, and between 0 and 1.5 h^−1^ for $b_{MR}$ and $b_{ED}$, with average $\overline{{b_{MD} }} = 1.64 \pm 2.3 \,{\text{h}}^{ - 1}$, $\overline{{b_{MR} }} = 0.78 \pm 0.77 {\text{h}}^{ - 1}$, $\overline{{b_{ER} }} = 2.19 \pm 3.2 {\text{h}}^{ - 1}$, $\overline{{b_{ED} }} = 1.2 \pm 1.6 {\text{h}}^{ - 1}$ (mean ± standard deviation). The mean ± standard deviation of the magnitude of the four rate constants from all flies tested in constant darkness is $1.5 \pm 2.0 {\text{h}}^{ - 1}$. Lastly, linear fits of the $T_{M\left( E \right)}$ versus $T_{0}$ data reveal $T_{M} (T_{0} ) \approx 0.19T_{0}$ and $T_{E} (T_{0} ) \approx 0.29T_{0}$ (${\text{Adj }}R^{2} = 0.90$ and $0.95$, respectively) (Fig. 5a, bottom panels).

Based on the observed independence of the *b* parameters and the linear dependence of the $T_{M(E)}$ parameters on the circadian period, we argued using the model that the average amplitude of daily activity must also increase with $T_{0}$ (Fig. 5b). To test this prediction, we measured for each fly an activity amplitude, h, by averaging M and E peak heights in each recording. A plot of h vs. $T_{0}$ shows that amplitude of activity indeed increases with lengthening of the circadian period (Fig. 5c). The form of our model function $F(t)$ suggests that h and $T_{0}$ can be related according to ${\text{h}} = c(1 - e^{{ - kT_{0} }} )$, which in the linear approximation becomes ${\text{h}} \approx CT_{0}$, with *c*, *k* and $C$ as fit parameters. Due to the large scatter in our data, the simpler linear expression is statistically favored over the exponential function, yielding ${\text{h(}}T_{0} )\approx 1.4T_{0}$ (Adj $R^{2} = 0.85$). The surprisingly good agreement between the data and the prediction of our simple model further validates the mathematical description of fly locomotion pattern proposed in this work.

## Discussion

While a large number of studies have exploited rhythmic patterns in fruit fly activity to query the status of the circadian clock, the broader question of what principal endogenous systems other than the clock shape these patterns, remain poorly understood. Here we attempt to fill this gap by formulating a simple mathematical model that accommodates the diverse locomotion patterns exhibited by *Drosophila* over the time-scale of multiple days. The model has two major components: an oscillatory component characterized by a single period and an exponential component defined by four rate constants. We show that these components are together sufficient to explain prominent features in both the power spectra and the time series of fly locomotor recordings. Using the model, we show that the position and height of over 88 % of locomotor spectral peaks can be accounted for as harmonics of circadian rhythms. We use this finding as evidence to support the view that in flies daily locomotor rhythms receive negligible input from endogenous ultradian clocks. Using the proposed model, we additionally extract values of the exponential rate constants in a variety of flies. We propose that these rate constants could represent kinetics of neurobiological processes that, such as the secretion of neuropeptides, have been implicated in modulating behavior. We suggest our data and model-based predictions open up fly behavioral recordings to more rigorous analysis.

Ultradian and circadian clocks are known to co-exist in animals but the extent of interactions among the two classes of endogenous oscillators is still unresolved. Previous work on this issue outlines three possible relationships between circadian and ultradian rhythms: i) a circadian rhythm is produced by the coupling of ultradian oscillators, and ultradian peaks seen in power spectra are components that break down from the circadian clock [19–21, 42]; ii) ultradian and circadian oscillators exist independently in the organism [15, 21]; and iii) ultradian rhythms come from the desynchronization of a population of circadian oscillators [21]. Using our model we can address the first two hypotheses. We have shown that multiple periodicities in power spectra result from a non-sinusoidal shape of *Drosophila* locomotion and appear at harmonics of the circadian rhythm (Fig. 3a, b). Since nearly all peaks in our fly power spectra come from circadian rhythms (Fig. 3c; Additional file 1: Figs. S2–S5), we suggest the first hypothesis, which states that circadian rhythms result from the coupling of ultradian oscillators, is unlikely. The assumption of the second hypothesis, that circadian and ultradian oscillators coexist, is possible; however we have shown that more than 88 % of peaks that stably appear between 2 and 35 h in the power spectra of fly locomotion result from the circadian clock. Our attempts to detect intermittent ultradian activity revealed such behavior in <5 % of the total recording time (Additional file 1: Fig. S7). These results therefore indicate that there are no robust ultradian oscillators with periods in this range detectable in fly activity. Our data are at odds with claims made by several previous examinations of locomotion [17, 19–21, 43]. The previous studies missed the harmonics likely due to a combination of disregarding the non-sinusoidal pattern of the raw time series, the inappropriate use of digital filters and the lack of proper statistical metrics. Our data, together with bona fide ultradian periodicities discussed in other behavioral contexts such as male courtship song [44, 45] or calcium oscillations in neurons [46], suggest that second hypothesis is most likely, and that there is minimal cross-talk between the two types of oscillators, at least at the level of behavioral output. In brief, our results contradict the previously espoused role of multiple ultradian oscillators shaping locomotion. Instead, the data support a parsimonious model in which a single circadian oscillator regulates all rhythmic modulations of fly locomotion.

Data on ultradian rhythms reported in locomotion of mammals can also be considered in relation to the circadian clock. Studies that addressed this question in Siberian hamsters and common voles concluded that the ultradian rhythms are generated independently of the circadian clock [15, 16]. Autonomy of mammalian ultradian rhythms was further affirmed by a recent work in mice that identified dopamine as a major regulator of and, possibly, the source of these ~4 h oscillations [18]. These previous data from mammals raises the question of why *Drosophila* locomotion does not exhibit robust ultradian oscillations. Our model, which incorporates only circadian rhythms, reliably predicts spectral peak heights and positions in rhythmic flies of different genotypes and genetic backgrounds and shows an absence of rhythms in circadian arrhythmic animals. Compared to mammals, the fly data suggest that if there is an ultradian signal that feeds into locomotion, it is either much weaker or has a period ≪2 h where our model does not strictly apply.

Beyond addressing the rhythmic component of locomotion, our model also captures the general temporal shape of fly locomotion. Despite widespread use of these time series in fly circadian studies, to our knowledge, proposed $F(t)$ is the first description of the multi-day data in mathematical terms. In our mathematical model, the various shapes of these time series are quantified in terms of four exponential rate constants, $b_{MD} - b_{ED}$, that control the slopes around the morning and evening activity peaks and the parameters $T_{M} - T_{E}$, that control the widths of these peaks. Although one may assume that all model parameters should scale with the circadian period, our measurements reveal that while $T_{M}$ and $T_{E}$ increase with the circadian period, the exponents $b_{MD} ,\,b_{MR}$,$b_{ER}$, and $b_{ED}$ intriguingly do not (Fig. 5a). Independence of the rates from the circadian period indicates that mutations in *per* and *tim* do not affect the rate constants $b_{MD} - b_{ED}$, suggesting that the rate constants possibly represent clock-autonomous biological processes. This observation together with available biochemical data, leads us to hypothesize that the rate constants could represent processes like accumulation, release or degradation of neuropeptides, particularly those that are involved in the clock neuronal network. Neuropeptides pigment dispersing factor (PDF), ion transport peptide (ITP) and neuropeptide F (NPF) modify M and E clock neurons and influence locomotor patterns [34, 47, 48]. The abundance of these neuropeptides, in turn, are modulated by the clock [34, 49]. But their rates of accumulation in neurons, subsequent secretion, and degradation are presumably controlled by cell-biological machinery whose rate-limiting steps are set independently of the circadian program [50]. Therefore, we propose the $b$ rate constants could represent average measures of some of these kinetic steps. Specifically, since PDF controls morning anticipation, which in our model is shaped by $b_{MD}$ and $b_{MR}$, we suggest these parameters may reflect kinetic steps related to PDF [33]. Similarly, because ITP reduces nocturnal activity and enhances diurnal evening activity, its kinetics may contribute to exponents $b_{ER}$ and $b_{ED}$ which form the E peak in our model [34]. Regarding temporal modulation of the levels of the neuropeptides, our model suggests that it likely results from the clock periodically initiating and terminating the key kinetic steps. In circadian rhythms, neuropeptide activity has so far been accessible largely through biochemical assays. Our analysis using $F(t)$ may have established a potential link to the relevant neuropeptide kinetics through the behavioral rhythms that these neuropeptides are thought to modulate.

Alternatively, the model exponents could represent signals that feed into locomotor circuits from other endogenous systems such as those controlling fly sleep–wake states. Daily temporal patterns in sleep and activity in flies, like in mammals, are coordinated with major inputs from the circadian clock and the sleep homeostat [36]. Studies on human sleep patterns, in particular, have led to a model in which the sleep homeostat output is described as an exponentially varying signal [35]. In this model, the varying signal is the sleep electroencephalogram (EEG) power density that exponentially increases during daytime wakefulness and diminishes during nighttime sleep, producing a singular peak in the signal in the early evening. The average rate constants associated with the increase and decrease of EEG power are ~0.06 and ~0.24 h^−1^, respectively. Interestingly, these are well within the range of values we find for the $b$ parameters. Although output of the fly sleep homeostat has yet to be defined, it is reasonable to assume that its architecture may resemble that of the human homeostat given the substantial mechanistic overlap already uncovered between fly and human sleep [36]. Together, these similarities are consistent with a hypothesis in which the rate constants extracted from fly locomotion could be representative of the fly sleep homeostat.

**Fig. 6:**
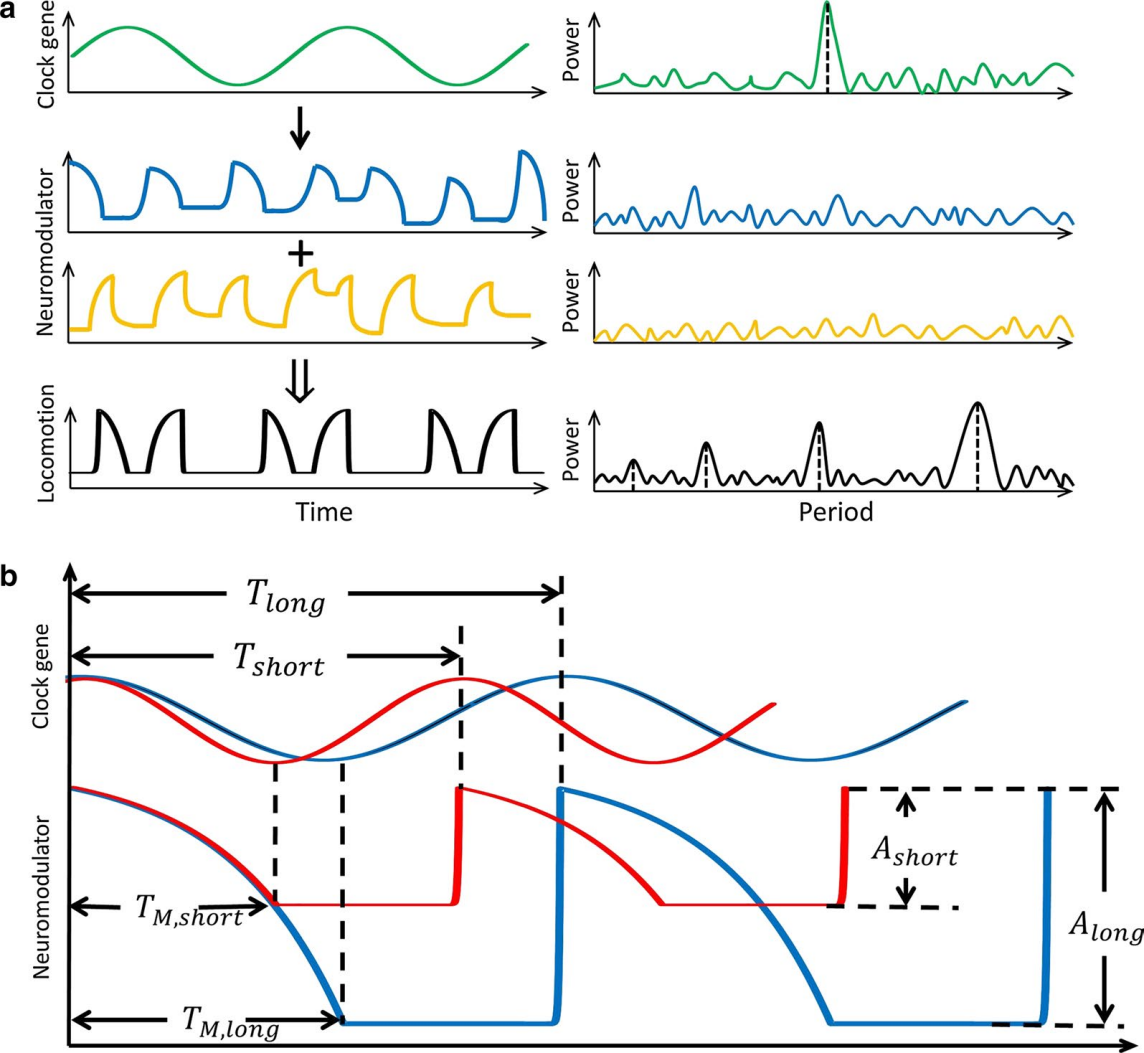
Our results make quantitative predictions about biochemical signals that may shape fly locomotor patterns. **a** Cartoon showing circadian genes and their products oscillate in a sinusoidal fashion (*top row*, *left*) and produce a power spectrum with a single peak (*top row*, *right*). We propose that efferent signal from the clock impinges on exponential processes that turn on/off stochastically in time. An example may be neuromodulator accumulation and release (*middle rows*, *left*). Without circadian regulation, the exponential process should result in noisy power spectra (*middle rows*, *right*). Integration of the sinusoidal with the exponential processes together with other signals (not shown) result in the observed shape of fly locomotion (*bottom row*, *left*). The resulting time series has multiple peaks in power spectrum by virtue of its non-sinusoidal shape (*bottom row*, *right*). **b** We suggest that the exponential processes that may underlie the observed shape in locomotion are temporally gated by the circadian clock. If so, then changing rhythm of the clock should cause corresponding changes both in the oscillatory period and the peak–peak amplitude of the gated signal. Shortening period of the clock ($T_{long} \to T_{short}$) predicts speeding up of the periodic exponential process and decrease of its amplitude of oscillation ($A_{long} \to A_{short}$). In these cartoons, it is assumed that the rate at which the exponential process occurs, for instance, the rate of neuromodulator release, is not affected by the circadian clock speed. To underscore the constant rate, the first decay phase of the fast and slow exponential waves are overlaid

Our mathematical model also permits a number of general predictions to be made about candidate neurobiological processes that may underlie our model parameters (Fig. 6). First, we propose that the candidate process can be described, for simplicity, in terms of linear or exponential growth-decay kinetics. Second, the processes are presumably sporadic when averaged over many neurons. Their random nature would imply they have noisy power spectra (Fig. 6a, middle row) and considering the processes are key modulators of behavior, their average stochastic nature should result in arrhythmic behavior in the absence of the circadian clock (Fig. 3c). Third, we suggest that the underlying processes have kinetics on the time-scale of tens of minutes. This constraint is based on the magnitude of the model rate constants, which are ~1.5 h^−1^ on average, suggesting that the processes they represent persist for ~40 min. At the molecular level, this is a long time-scale and suggests that neurotransmitter activity or electrical pulses, which also have exponential kinetics but act on the time-scale of seconds or less, are unlikely to be direct contributors to the exponents in $F(t)$. However, neuropeptide half-life in the brain and accumulation in synaptic boutons happen over span of ~30 min, making these substrates attractive candidates in our model [50]. Lastly, we suggest that on the time-scale of hours the circadian clock is likely the sole pacemaker (Fig. 6a, top row) to temporally gate the relevant neurobiological processes. The circadian gating imposes rhythm on otherwise stochastic processes and when integrated with additional downstream signals, ultimately produces rhythmic patterns in locomotion (Fig. 6a, bottom row). Related to the circadian gating are two additional features that the hypothetical processes might display (Fig. 6b). We suggest that changes in the period of the clock may cause not only parallel changes in the rhythm of the processes but, importantly, also changes in the amplitude of their oscillation. For instance, if $b_{MD}$ and $b_{MR}$ are indeed related to PDF activity, our model predicts that compared to *per*^L^ animals, in *per*^S^ animals both the period and the peak–peak amplitude of PDF oscillation is smaller. This prediction is based on our assumption that such changes in the amplitude of the underlying processes likely causes the correlation observed in our data between the average locomotor amplitude and the circadian period, which is predicted surprisingly well by our model (Fig. 4b, c). Together, these characterizations should facilitate experimental identification of the key neurobiological processes that our model parameters represent.

## Conclusions

In summary, this work initiates a more mathematical dissection than is currently available of the temporal shape of fly locomotor recordings. We point out that such analysis can lead to experimentally verifiable predictions of what major biological processes regulate daily manifestations of behavior. As examples of such candidates, we propose accumulation and degradation of neuropeptides implicated in controlling sleep-activity in flies and suggest these kinetic steps are gated principally by the circadian clock. Critically, our mathematical model now opens up the recordings to quantitative scrutiny towards clearer mechanistic understanding of daily rhythmic behaviors in *Drosophila* and other organisms.

## Methods

### Fly strains and recording

The following fly strains were used in this study: *Canton*-*S*, *yw*, *per*^0^, *per*^*S*^, *per*^*L*^ and *tim*^*UL*^. All flies were raised on standard Drosophila medium (corn meal, agar, malt, yeast). Only 2–5 day old male flies were used in the experiments. In all experiments *Canton*-*S* flies were used as wild type control, except in constant light experiments where *yw* flies were used, since *per*^0^ flies have *yw* background.

Locomotion was recorded using activity monitors (DAM5 TriKinetics Inc., MA). Monitors were placed in an environmental chamber (Percival Scientific DR36VL, max light intensity 4000 lx) maintained at 25 degrees Celsius and 70–80 % relative humidity. In all experiments flies were first entrained in 12-h light/12-h dark conditions for 2–3 days. In light/dark experiments, after entrainment locomotion was measured in the 12 h light/12 h dark day with 20 s binning for 5 days. In constant darkness experiments, activity of *Canton*-*S*, *per*^0^, *per*^*S*^, *per*^*L*^ and *tim*^*UL*^ flies was measured with 20 s binning for 5–7 days. The first day of constant darkness after entrainment was not used in the analysis.

### Spectral analysis

Data from monitors were visualized and processed with custom written Matlab (MathWorks Inc., MA) scripts. Power spectra of fly locomotion were calculated using two methods: maximum entropy spectral analysis (MESA) [51] and Lomb–Scargle periodogram (LS) (http://www.mathworks.com/matlabcentral/fileexchange/22215-lomb-normalized-periodogram). The power spectra produced by LS are normalized by the data variance. MESA uses an autoregressive model as an approximation for time series. In this model, a point at time *t* is given as a linear combination of previous *m* data points, where *m* is the order of the model. Coefficients of the linear approximation form a filter and are used to generate the power spectrum of the time series using the Andersen algorithm [52]. Although MESA provides high resolution even with noisy signals typical in fly locomotion, it does not have a convenient significance test for spectral peaks. The Lomb Scargle method is a slightly modified classic periodogram and was developed to detect weak rhythms in noisy data. LS is similar to the Fourier analysis method, but with better resolution and an easily computable statistical metric for noise discrimination. Additionally, since the LS method is similar to the classic periodogram, its results can be directly compared to the Fourier transform. These advantages make LS particularly applicable in our studies since we seek to distinguish real periodicities from noise and use analytically derived power spectra to determine model parameters.

### Power spectra fit and determination of model parameters

The analytical expression for peak heights $H(T_{n} )$ in the power spectrum was obtained using Mathematica (Wolfram Research, IL), while fitting was performed using Matlab. To get the expression for the peak heights $H(T_{n} )$ we calculated the Fourier transform of the model function $F(t)$ as a periodic signal (see Additional file 1):

$$ \tilde{F}(T_{n} ) = \frac{1}{{T_{0} }}\mathop \int \limits_{0}^{{T_{0} }} F(t) e^{{i\frac{2\pi n}{{T_{\small 0} }}t}} dt, $$

where $T_{0}$ is the primary period in the model and $T_{n} = T_{0} /n$, with $n = 1,2,3, \ldots,$ are harmonics. The square of this Fourier transform gives values of $H\left( {T_{n} } \right)$ [Additional file 1: equations (5)–(8)].

Model parameters were obtained in the following way. First, we determined power spectrum (PS) of the data using Lomb–Scargle periodogram. The PS was used to determine the primary period $T_{0}$ of locomotion. In the DD data, the period was found from peak at the circadian frequency. For flies measured in LD, the 12 h peak is typically the strongest, therefore value for $T_{0}$ was set by doubling the period of the second harmonic. Data were next binned into 20 min bins so the M and E peaks are better visualized to manually determine parameters $T_{M}$ and $T_{E}$ which correspond to the morning and evening peak widths. Initial values of the $b$ parameters were obtained from fitting the 20-min binned data with the model function $F(t)$. In order to determine the final set of parameters, we fitted the PS between 2 and 35 h with the obtained analytical expression [Additional file 1: equation (8)], using the initial parameter values as a first guess. To accelerate the fitting process, we restricted to ±60 h^−1^ the algorithm’s search for optimal rate constants; this limit was reached only occasionally. In our analysis we did not use power spectra for periods lower than 2 h, since in this range the PS typically does not have peaks above the *p* = 0.005 threshold.

### Fit accuracy analysis

To determine how well our model interprets the data power spectra, we calculated what fraction of the spectra is predicted by the analytical expression. The fraction was determined as the ratio of area of peaks in the data power spectra predicted by the model ($A_{predicted}$) to the total area of the spectra ($A_{total}$): $Accuracy = \frac{{A_{predicted} }}{{A_{total} }} \times 100\,\%$. The accuracy was determined for all flies that were used in our work. On average, our model was able to interpret more than 92 % of the data power spectra. Since this analysis is biased towards larger peaks, we also analyzed accuracy by calculating what fraction of peaks is interpreted by the model. We compared number of peaks in the data between 2 and 35 h, that are higher than *p* = 0.005, to number of peaks predicted by the model. This approach revealed that our model predicts ~88 % of peaks in the data power spectra.

In order to check fit accuracy we compared individual peak heights predicted by the analytical expression [Additional file 1: equation (8)] to the actual peak heights in the data power spectra. The fit accuracy was analyzed for the wild type flies measured both in light/dark and constant dark conditions. For each peak in the power spectra we calculated the percent error (*P.E.*) from the equation: $P.E. = \frac{{P_{data} - P_{expression} }}{{P_{data} }} \times 100\,\% ,$ where $P_{data}$ is a peak height in the data power spectra and $P_{expression}$ is a peak height predicted by the analytical expression. The average percent error was calculated for each peak for different light conditions. On average, the error was <10 %.

### Signal filtering

In our work, we did not filter the raw data. Low pass Butterworth filtering was employed in some previous studies seeking ultradian rhythms in fruit fly and mouse locomotion. However, filtering can dramatically affect the power spectrum. For instance, when a low-pass Butterworth filter was used on simulated white noise, we found that filtering a noisy aperiodic time series can lead to detection of spurious peaks (Additional file 1: Fig. S9 and associated text in Additional file).

## Additional file


10.1186/s12868-016-0248-9 Supplementary material for the main manuscript. The file contains additional data that support our findings, detailed mathematical derivation of the model power spectrum, and mathematical analysis of effects of the Dirichlet kernel and Butterworth filter on power spectra.

## Abbreviations

LD: 12 h light/12 h dark conditions
LL: constant light conditions
DD: constant darkness conditions
DAM: Drosophila Activity Monitor
PDF: pigment dispersing factor
ITP: ion transport peptide
NPF: neuropeptide F
EEG: electroencephalogram
MESA: maximum entropy spectral analysis
LS: Lomb–Scargle periodogram
