# Supplementary material for: A mathematical model provides mechanistic links to temporal patterns in Drosophila daily activity
Source: BMC Neurosci. 2016 Apr 18;17:14. doi: 10.1186/s12868-016-0248-9 (PMC4835852; doi:10.1186/s12868-016-0248-9)
Supplement: Supplementary file 1 — 10.1186/s12868-016-0248-9 Supplementary material for the main manuscript. The file contains additional data that support our findings, detailed mathematical derivation of the model power spectrum, and mathematical analysis of effects of the Dirichlet kernel and Butterworth filter on power spectra. [file 12868_2016_248_MOESM1_ESM.pdf]

Supplementary Materials

Power spectra of different waveforms

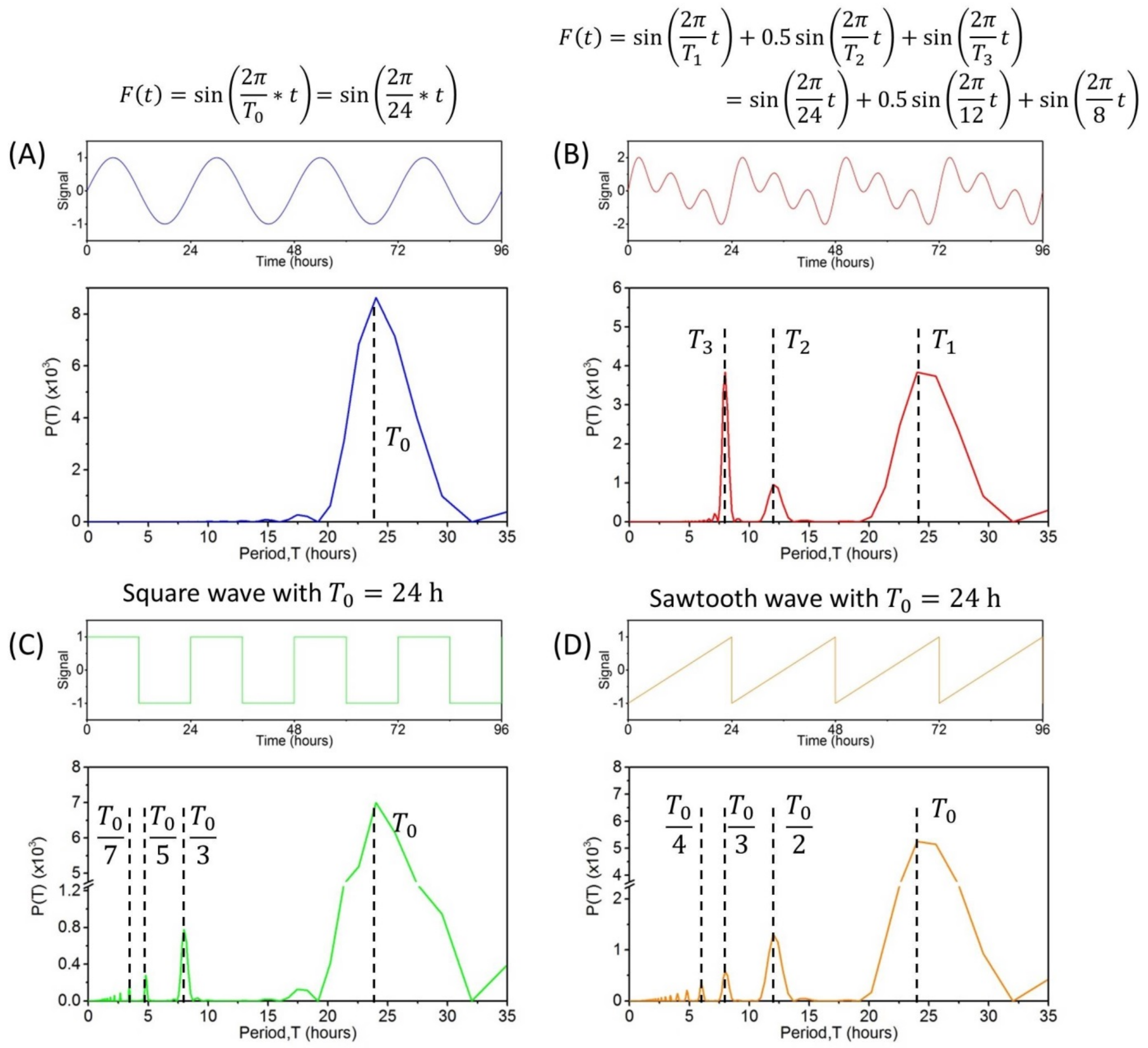

Figure S1. Shape of the activity waveform is a strong determinant of power spectra. (A) Power spectrum $P(T)$ of a sine wave with a single period shows a single peak while (B) that with three distinct periods shows three peaks with peak heights reflecting the relative amplitude of each sine wave in the signal. (C) In contrast, a square wave with a single natural period T_0 produces

$P(T)$ with peaks at $T_0/(2n-1)$, while (D) a sawtooth wave with period T_0 produces peaks at T_0/n, all odd and even harmonics of the fundamental. $n = 1,2,3$ ... positive integers.

Analysis of arrhythmic mutants of Drosophila melanogaster

Locomotor activity of per^0 and clk^{Jrk} clock mutants was measured in constant darkness for 4-5 days after entrainment in 12 hour light/12 hour dark conditions for 2 days. Measurements from the first day in DD were not used for calculation of power spectra. Power spectra were calculated using the Lomb-Scargle method.

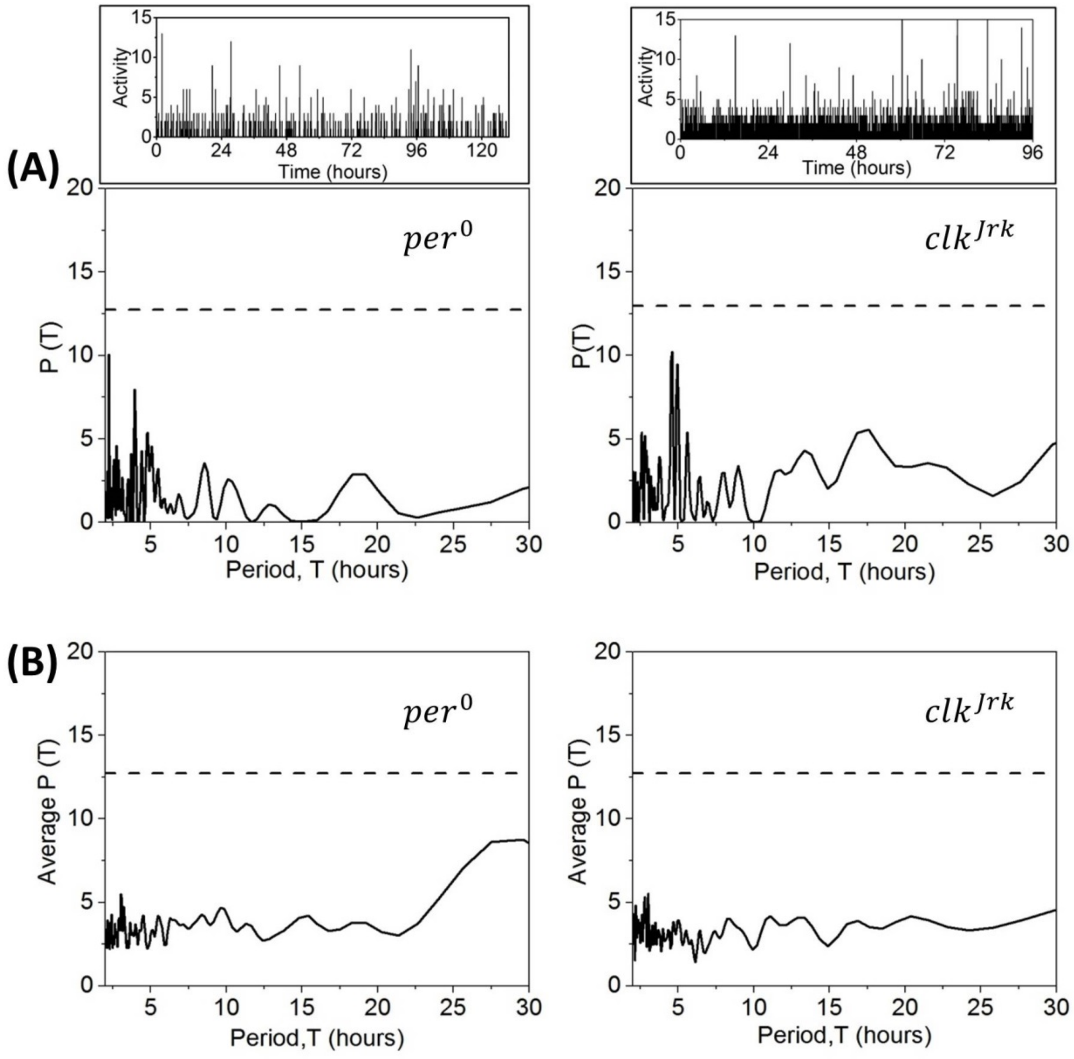

Figure S2. Analysis of clock null mutants reveals lack of rhythmicity across the ultradian-circadian range. (A) Power spectra of single per^0 and clk^{Jrk} flies show all features between 2 and 30 hours lie below the respective $p = 0.05$ (dashed line) statistical cut-off. Locomotor data of the flies shown in boxes (top row). (B) Population-averaged power spectrum of per^0 and clk^{Jrk}. Average $P(T)$ was calculated from individual power spectra of 28 arrhythmic per^0 flies and 17 arrhythmic clk^{Jrk} flies measured in constant darkness. Dashed lines show significance level of 0.05.

Among the per^0 mutants (N=38), 28 flies did not show peaks higher than significance level of 0.05 between 2 and 35 hours in power spectra (Fig. S2). The remaining 10 flies showed weak periodicities in the circadian range. Among the clk^{Jrk} (N=23), 17 flies did not show any significant periodicity, while 6 flies showed moderate or weak circadian rhythms.

Additionally, we analyzed the clk^{AR} (N=28) mutant that was reported to be arrhythmic in DD [1]. However, in our analyses most flies were not arrhythmic. We divided these flies according to their $P(T)$ into three groups (Fig. S3): (1) arrhythmic flies (N=6), (2) flies that do not have a circadian peak, but show peaks for periods smaller than 24 hours (N=18), and (3) flies that have a circadian period (N=4). It is possible that this variety of behavior results partly from clk^{AR} flies having low levels of oscillating per and tim proteins [1, 2], since low levels of clock genes have been shown to be associated with abnormal rhythmic patterns [3]. Regardless, the observed peaks are several times smaller ($\lesssim 30$) than peaks in periodic data ($> 10^2$) of similar length.

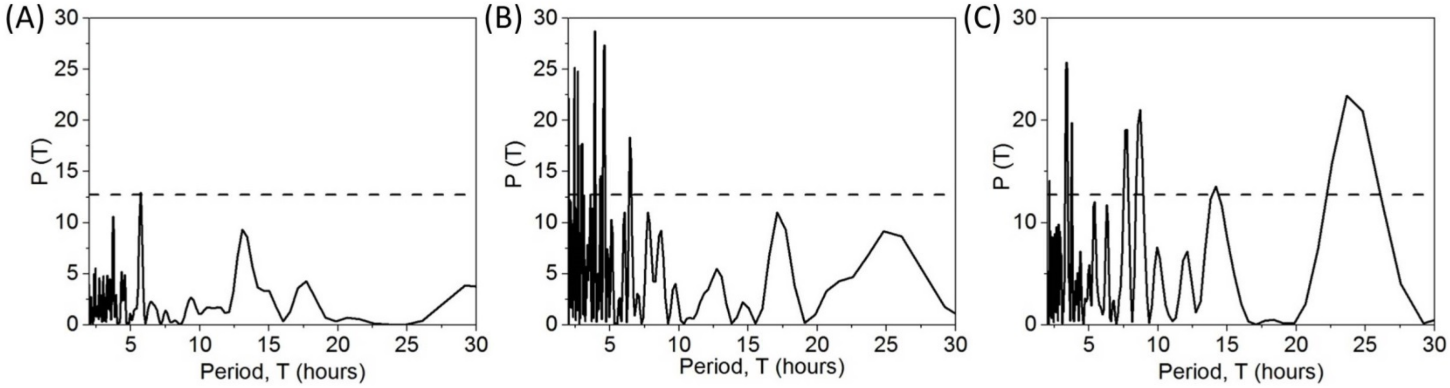

Figure S3. Population of clk^{AR} flies displays three types of rhythmicity (A) Power spectrum of an arrhythmic fly. (B) Power spectrum of a fly that does not have circadian rhythm, but shows multiple peaks for $T < 5$ hours. (C) Power spectrum of a clk^{AR} fly with strong circadian rhythm.

Shown power spectra are calculated from a 4 day recording in DD. Dashed horizontal line represents $p = 0.05$ confidence level.

To confirm results obtained by Lomb-Scargle periodogram, we next calculated power spectra of the above data using Maximum Entropy Spectral analysis method (MESA). Unlike Lomb-Scargle periodogram, MESA does not have a built in significance test, and so in order to determine statistical significance, we compared peak heights in the data power spectra to typical peak heights in the power spectra of random time series. Random time series simulations of the same length as data (N=17000) were obtained by averaging 1000 artificial data sets generated with the MATLAB function ‘randn.m’, which outputs values from the standard normal distribution. We found that the MESA-calculated peak heights from per^0 and the random series are comparable to each other and do not exceed 1 (Fig. S4). In contrast, typical peak height in the 2
MESA power spectrum of a periodic recording of similar length is ~10^3. Thus, results obtained by MESA are consistent with those from LS periodogram, both suggesting that null mutants of the circadian clock have no robust rhythms in their locomotion.

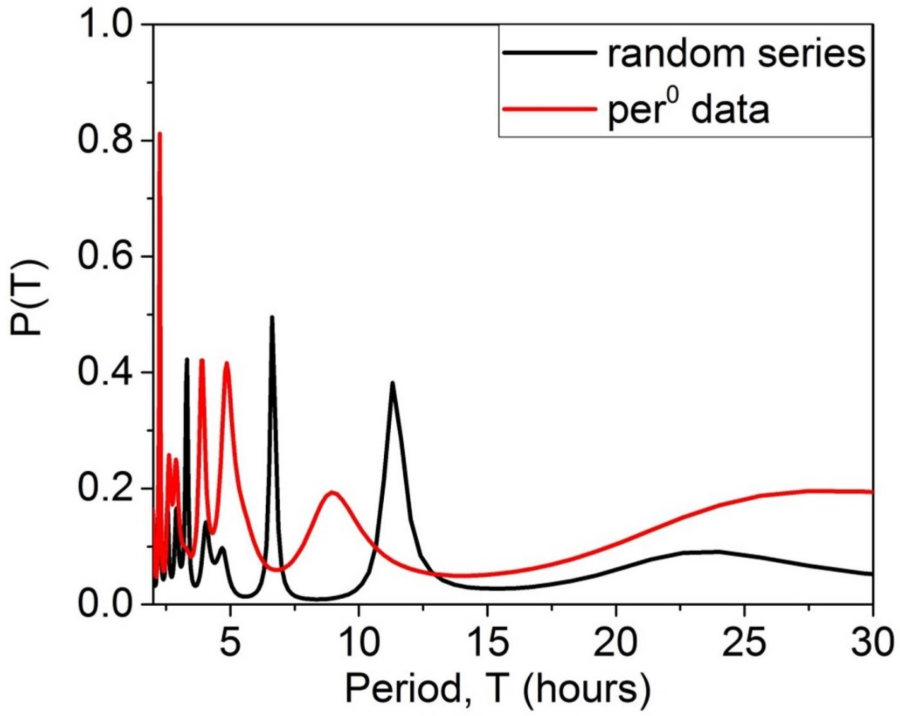

Figure S4. Features in the MESA power spectrum of a per^0 fly (red) are comparable to those of a computer generated random time series (black).

Analysis of wild type flies in constant light

Locomotor activity of *yw* flies (N=16) was measured in constant light (LL) for 6 days. Flies were first entrained in 12 hour light/12 hour dark for 1 day and subsequently placed in constant light. Measurements from the first 2 days of LL were excluded from analysis. Lomb Scargle periodograms of majority of the flies (N=13) show no statistically significant peaks at the $p = 0.05$ level (Fig. S5).

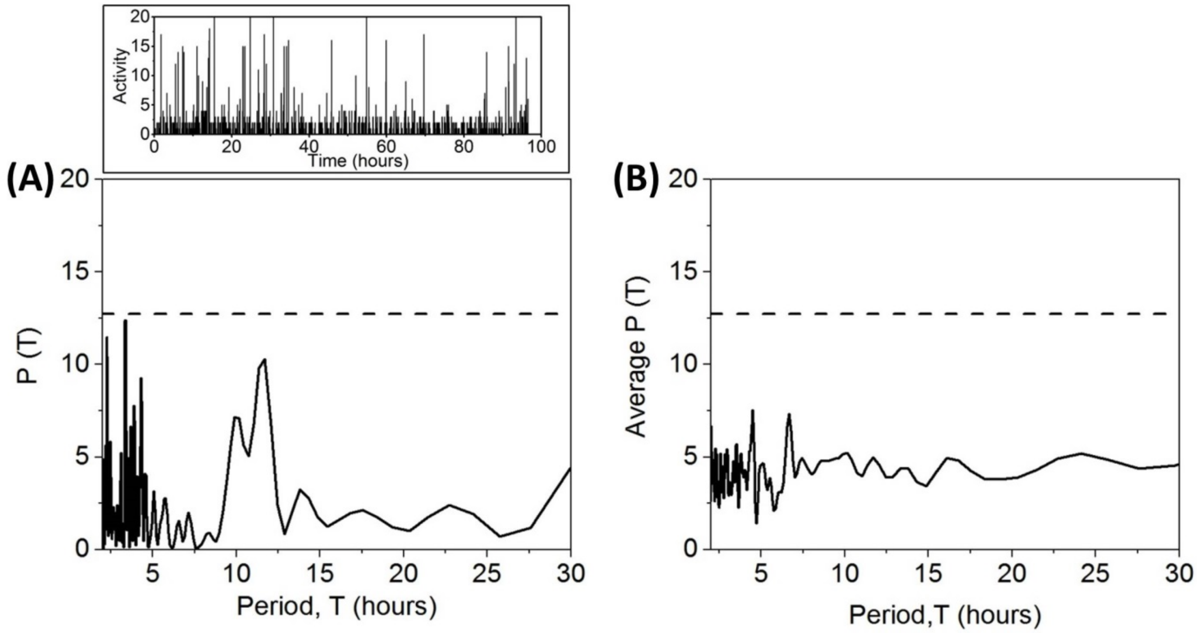

Figure S5. Analysis of *yw* flies in constant light. (A) Power spectrum of a single arrhythmic fly shows that the activity (box, top row) contains no rhythmicity with period between 2 and 30 hours. (B) Average power spectrum for the wild type flies. Average $P(T)$ was calculated from the power spectra of 13 arrhythmic flies measured in LL. Dashed line denotes $p = 0.05$ in both panels.

In addition to *yw*, we also examined *Canton-S* wild type flies (N=16) in constant light. Unlike *yw* flies > 80% of which were arrhythmic, *Canton-S* flies showed three types of activity in LL: (1) flies that have strong circadian rhythm (N=6), (2) flies without circadian rhythm, but with periodicities less than 24 hours (N=7), (3) flies that do not show any significant peaks in power spectra (N=3) (Fig.S6). Similar results were observed in Siberian hamsters after they were subjected to disruptive phase-shifting protocol [4]. The three types of behavioral pattern seen in LL activity likely result from differences in light sensitivity in the fly population.

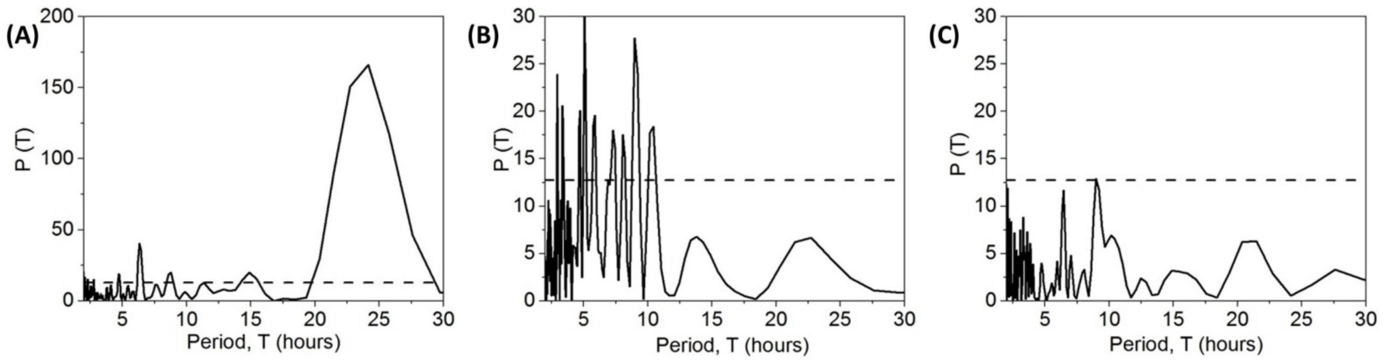

Figure S6. *Canton-S* wild type strain shows three types of activity in LL. (A) Power spectrum of a fly with strong circadian rhythm (B) Power spectrum of a fly with multiple peaks between 2 and 10 hours (C) Power spectrum of an arrhythmic fly. Dashed horizontal line in each panel denotes $p = 0.05$.

We have analyzed over 300 flies of different genotypes and backgrounds measured under various light conditions (LD, DD, LL). The mathematical framework of our model accommodates more than 85% of the flies examined thus far. Here it is important to reiterate that locomotion data in this work were collected with single infrared beam detectors, in accordance with the standard technique employed in fly circadian research. The lack of spatial resolution

inherent to this technique effectively ‘filters’ the locomotion recordings, thus, as we show in the section “Low –pass Butterworth filter”, artificially lowering variance in data and overestimating the statistical significance of features in the power spectra (Fig. S9). Therefore, we expect higher resolution locomotion data, such as those acquired with multiple beams, will likely show that the low- power peaks observed occasionally (see for example, Fig. S3 B,C; Fig. S6 B) can also be rejected on statistical grounds.

Detection of transient oscillations

We subjected yw in LL and per^0 in DD to additional scrutiny to detect oscillations that may persist for only portions of the multiday-long time series. Animals that appeared arrhythmic over long time scale in the above analyses (yw, N=13; per^0 ,N=28) were individually examined for presence of ultradian periodicities between 2 and 6 hours by calculating power spectra of a 12 hour moving window with left edge at time t_0 (Fig. S7A). We initialized t_0 at 0 and incremented its value in 1 hour steps. For each window, we determined spectral peaks between 2 and 6 hours above the p=0.005 threshold. The observed oscillations were plotted versus t_0 for individual flies (example in Fig. S7B). This analysis revealed 50% of flies do not show any such transient oscillations. The remaining 50% of the flies that did show bursts of oscillations did so, on average, for only ~9% of the length of time series. These statistics suggest the oscillations are very rare and when they occur, are unstable, leading us to consider them insignificant.

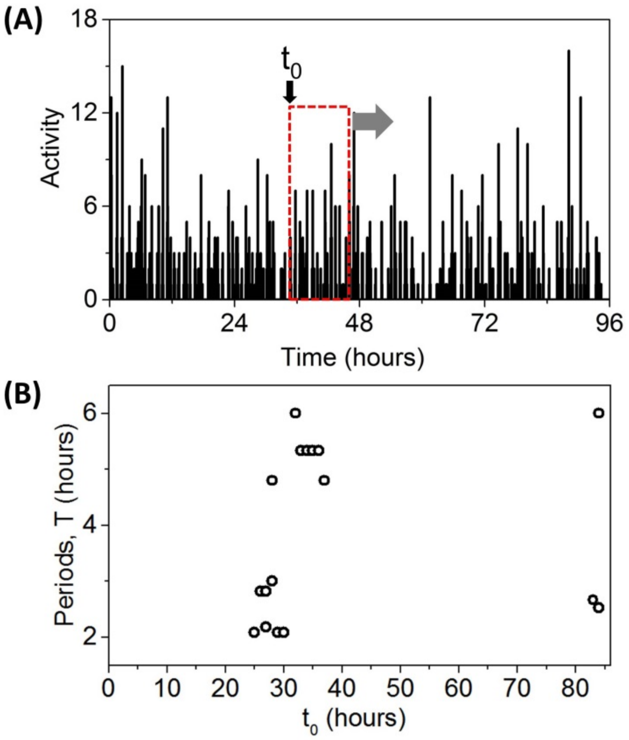

Figure S7. Arrhythmic flies were tested for the presence of short-lasting ultradian oscillations between 2 and 6 hours. (A) Power spectrum was calculated for 12 hour stretches of activity (shown with red dashed rectangle) starting from t_0. Single flies were analyzed with multiple t_0 starting from 0 with step 1 hour. (B) Plot of the observed peaks higher then *p*=0.005 versus t_0 for one fly. For bulk of the recording, the fly exhibits no rhythmic signal.

Power spectrum calculation

To obtain analytical expression for peak heights at harmonics of primary period we calculated Fourier transform of our model function using Mathematica. Model function $F(t)$:

$$F(t) = \begin{cases} \dfrac{e^{b_{MD}T_M} - e^{b_{MD}t}}{e^{b_{MD}T_M} - 1}, & 0 < t < T_M \\ \dfrac{e^{b_{MR}(t-T_M)} - 1}{e^{b_{MR}(T_0-T_M)}}, & T_M < t < T_0 \\ \dfrac{1 - e^{-b_{ER}\left(t-\frac{T_0}{2}-T_E\right)}}{1 - e^{-b_{ER}T_E}}, & \dfrac{T_0}{2} - T_E < t < \dfrac{T_0}{2} \\ e^{-b_{ED}\left(t-\frac{T_0}{2}\right)}, & \dfrac{T_0}{2} < t < T_0 \end{cases} \quad (1)$$

consists of four exponential terms, where the first two exponents form a morning peak and remaining two exponents form an evening peak. In Mathematica, $F(t)$ was defined as the sum of $g(t)$ and $f(t)$ as:

$$F(t) = f(t) + g(t),$$

where $f(t)$ forms a morning peak and contains first two exponential terms that are written using the Heaviside function $\theta(t)$:

$$f(t) = \left(\theta(t) - \theta(t - T_M)\right) \frac{e^{b_{MD}T_M} - e^{b_{MD}t}}{e^{b_{MD}T_M} - 1} + \left(\theta(t - T_M) - \theta(t - T_0)\right) \frac{e^{b_{MR}(t-T_M)} - 1}{e^{b_{MR}(T_0-T_M)}} \quad (2)$$

and $g(t)$ function forms an evening peak and contains the other two exponential terms:

$$g(t) = \left(\theta\left(t - \left(\frac{T_0}{2} - T_E\right)\right) - \theta\left(t - \frac{T_0}{2}\right)\right)\frac{1 - e^{-b_{ER}\left(t - \frac{T_0}{2} - T_E\right)}}{1 - e^{-b_{ER}T_E}} + \left(\theta\left(t - \frac{T_0}{2}\right) - \theta(t - T_0)\right)e^{-b_{ED}\left(t - \frac{T_0}{2}\right)} \quad (3)$$

The Fourier transform of $F(t)$ can be calculated as

$$\tilde{F}(\omega) = \frac{1}{2\pi}\int_{-\infty}^{+\infty} F(t)e^{i\omega t}dt$$

However, in our work we have a periodic function with primary period T_0 and we are interested only in solutions at harmonics of the period. Therefore, we used a simplified expression for Fourier transform that considers $F(t)$ as a periodic function over the length of one period:

$$\tilde{F}(T_n) = \frac{1}{T_0}\int_{0}^{T_0} F(t)e^{\frac{i2\pi n}{T_0}}dt \quad (4)$$

where $T_n = T_0/n$ with $n = \{1,2,3,\ldots\}$. The square of this Fourier transform gives the peak heights $H(T_n)$. Results of the above integral can be separated into real and imaginary terms, $Re(n)$ and $Im(n)$ respectively, to calculate the heights as:

$$H(T_n) = Re(n)^2 + Im(n)^2 \quad (5)$$

Here $Re(n)$ and $Im(n)$ are given by:

$$Re(n) = -\frac{1}{2}T_0\left(C_1 - A_1(n)\,cos\left(\frac{2T_M n\pi}{T_0}\right) - A_2(n)\,cos\left(\frac{2T_E n\pi}{T_0}\right)\right.$$

$$\left. + A_3(n)\,sin\left(\frac{2T_M n\pi}{T_0}\right) + A_4(n)\,sin\left(\frac{2T_E n\pi}{T_0}\right)\right) \tag{6}$$

$$Im(n) = \frac{1}{2}T_0\left(C_2 - A_3(n)\,cos\left(\frac{2T_M n\pi}{T_0}\right) + A_4(n)\,cos\left(\frac{2T_E n\pi}{T_0}\right)\right.$$

$$\left. - A_1(n)\,sin\left(\frac{2T_M n\pi}{T_0}\right) + A_2(n)\,sin\left(\frac{2T_E n\pi}{T_0}\right)\right) \tag{7}$$

with coefficients:

$$A_1(n) = \frac{2b_{MD}T_0e^{T_M b_{MD}}}{(-1+e^{T_M b_{MD}})(4n^2\pi^2 + b_{MD}^2T_0^2)} + \frac{2b_{MR}T_0e^{T_M b_{MR}}}{(e^{b_{MR}T_0} - e^{T_M b_{MR}})(4n^2\pi^2 + b_{MR}^2T_0^2)}$$

$$A_2 = (-1)^n \frac{2b_{ER}T_0e^{T_E b_{ER}}}{(-1+e^{T_E b_{ER}})(4n^2\pi^2 + b_{ER}^2T_0^2)}$$

$$A_3(n) = \frac{b_{MD}^2T_0^2e^{T_M b_{MD}}}{(-1+e^{T_M b_{MD}})\,n\pi\,(4n^2\pi^2 + b_{MD}^2T_0^2)} + \frac{b_{MR}^2T_0^2e^{T_M b_{MR}}}{(e^{b_{MR}T_0} - e^{T_M b_{MR}})n\pi\,(4n^2\pi^2 + b_{MR}^2T_0^2)}$$

$$A_4 = (-1)^n \frac{b_{ER}^2T_0^2e^{T_E b_{ER}}}{(-1+e^{T_E b_{ER}})\,n\pi\,(4n^2\pi^2 + b_{ER}^2T_0^2)}$$

$$C_1(n) = \frac{2b_{MD}T_0}{(-1+e^{T_M b_{MD}})(4n^2\pi^2 + b_{MD}^2T_0^2)} + \frac{2b_{MR}T_0e^{b_{MR}T_0}}{(e^{b_{MR}T_0} - e^{T_M b_{MR}})(4n^2\pi^2 + b_{MR}^2T_0^2)}$$

$$+ (-1)^n \frac{2b_{ER}T_0}{(-1+e^{T_E b_{ER}})(4n^2\pi^2 + b_{ER}^2T_0^2)} + (-1)^n \frac{2b_{ED}T_0}{(4n^2\pi^2 + b_{ED}^2T_0^2)}$$

$$- \frac{2b_{ED}T_0e^{-\frac{1}{2}b_{ED}T_0}}{(4n^2\pi^2 + b_{ED}^2T_0^2)}$$

$$C_2(n) = \frac{e^{T_M b_{MR}}}{(e^{b_{MR}T_0} - e^{T_M b_{MR}})n\pi} + \frac{e^{T_M b_{MD}}}{(-1 + e^{T_M b_{MD}})\, n\pi} - \frac{4\, n\, \pi}{(-1 + e^{T_M b_{MD}})(4n^2\pi^2 + b_{MD}^2 T_0^2)}$$

$$- \frac{4\, n\, \pi\, e^{b_{MR}T_0}}{(e^{b_{MR}T_0} - e^{T_M b_{MR}})(4n^2\pi^2 + b_{MR}^2 T_0^2)} - (-1)^n \frac{e^{T_E b_{ER}}}{(-1 + e^{T_E b_{ER}})\, n\pi}$$

$$+ (-1)^n \frac{4\, n\, \pi}{(-1 + e^{T_E b_{ER}})\ (4n^2\pi^2 + b_{ER}^2 T_0^2)} - \frac{4\, n\, \pi\, e^{-\frac{1}{2} b_{ED} T_0}}{(4n^2\pi^2 + b_{ED}^2 T_0^2)}$$

$$+ (-1)^n \frac{4\, n\, \pi}{(4n^2\pi^2 + b_{ED}^2 T_0^2)}$$

The peaks heights in power spectra are proportional to the number of cycles of given period in time series (N) and square of the average amplitude in the time series (h). Therefore the final equation for $H(T_n)$ is

$$H(T_n) = \alpha h^2 N(Re(n)^2 + Im(n)^2), \tag{8}$$

where α is a fit parameter that shows values from 0.3×10^{-3} to 3×10^{-3} for our data. For a given time series α is a rough measure of noise in the data. In this form, $H(T_n)$ was encoded into locally written Matlab program to perform fit of fly activity power spectra (see Methods for additional details).

Power spectrum for small period values

For $T_n \ll 2\pi/b$, where b is the largest rate, we have $4n^2\pi^2 \gg b^2T_0^2$ for all b's. In this limit, we can simplify expression of $H(T_n)$ by dropping all $b^2T_0^2$ terms. Then for the spectrum we have:

$$H(T_n) = Nh^2\alpha(Re(n)^2 + Im(n)^2), \text{ with}$$

$$Re(n) \approx -\frac{T_0^2}{4n^2\pi^2}\Bigg((-1)^n b_{ED} - b_{ED}e^{-\frac{1}{2}b_{ED}T_0} + \frac{b_{MD}}{(-1+e^{T_M b_{MD}})} + \frac{b_{MR}e^{b_{MR}T_0}}{(e^{b_{MR}T_0} - e^{T_M b_{MR}})}$$

$$+ \frac{(-1)^n\, b_{ER}}{(-1+e^{T_E b_{ER}})} + \left(-\frac{b_{MD}e^{T_M b_{MD}}}{(-1+e^{T_M b_{MD}})} + \frac{b_{MR}e^{T_M b_{MR}}}{(e^{b_{MR}T_0} - e^{T_M b_{MR}})}\right) cos\left(\frac{2T_M n\pi}{T_0}\right)$$

$$- (-1)^n \frac{b_{ER}e^{T_E b_{ER}}}{(-1+e^{T_E b_{ER}})} cos\left(\frac{2T_E n\pi}{T_0}\right)\Bigg)$$

$$Im(n) \approx \frac{T_0}{4\,n^2\pi^2}\Bigg(-2\,e^{-\frac{1}{2}b_{ED}T_0} n\,\pi - \left(\frac{b_{MD}e^{T_M b_{MD}}}{(-1+e^{T_M b_{MD}})} + \frac{b_{MR}e^{T_M b_{MR}}}{(e^{b_{MR}T_0} - e^{T_M b_{MR}})}\right) T_0\, sin\left(\frac{2T_M n\pi}{T_0}\right)$$

$$+ (-1)^n \frac{b_{ER}e^{T_E b_{ER}}}{(-1+e^{T_E b_{ER}})} sin\left(\frac{2T_E n\pi}{T_0}\right)\Bigg)$$

This equation predicts $H(T_n)$ to go as $1/n^4$, and since $T_n = T_0/n$, the peak heights should go as T_n^4 (Fig.4). For most of our data the highest rate, typically b_{MD} or b_{ER}, has value $\sim 1.7/\text{h}$, and we therefore expect this trend in the $H(T_n)$ for all $T_n \lesssim 2$ h.

Dirichlet kernel

Although the analytical expression for $H(T_n)$ produces strong peaks at harmonics of the fundamental period, it does not account for the nearby smaller peaks (for example, see arrows in Fig. 2C). In the analysis of activity data, one always works with discrete time series of finite length and one of the features in the periodogram of such limited time series is the presence of additional small peaks that appear due to the Dirichlet kernel.

Assume that we are given an infinite time series y_n. Then we pick short section of it:

$$x_n = \begin{cases} y_n, & 0 \le n \le N \\ 0, & otherwise \end{cases} \tag{9}$$

We can write operation of getting x_n from y_n as a multiplication of time series y_n on square pulse w_n:

$$x_n = y_n \cdot w_n, \text{ where } w_n = \begin{cases} 1, & 0 \leq n \leq N \\ 0, & otherwise \end{cases} \tag{10}$$

Therefore, for Fourier transform of x_n we will have [5]:

$$X(f) = \frac{1}{2\pi}\{Y(f) * W(f)\}, \tag{11}$$

where $Y(f)$ and $W(f)$ are Fourier transform of y_n and w_n, respectively, and $\{Y(f) * W(f)\}$ is a convolution of the two series. The Fourier transform $W(f)$ can be calculated as:

$$W(f) = \sum_{n=0}^{N-1} e^{-i2\pi fn} = \frac{1 - e^{-i2\pi fN}}{1 - e^{-i2\pi f}} = \frac{\sin(\frac{1}{2}2\pi fN)}{\sin\left(\frac{1}{2}2\pi f\right)} \; e^{-i\frac{1}{2}2\pi f(N-1)} \tag{12}$$

The function

$$D_N = \frac{\sin(\frac{1}{2}2\pi fN)}{\sin\left(\frac{1}{2}2\pi f\right)} \tag{13}$$

is a Dirichlet kernel. Square of the Dirichlet kernel has a strong peak at $f = 0$ and multiple small peaks on both sides of the $f = 0$ peak (Fig. S8 A). Number and position of the small peaks are determined by the order *N* of the kernel. Effect of the Dirichlet kernel is clearly seen in simulated $F(t)$ (eq.(1)) of different lengths (Fig. S8 B). While the positions of the major peaks are insensitive to *N*, the side peaks change position and increase in number with increasing *N*.

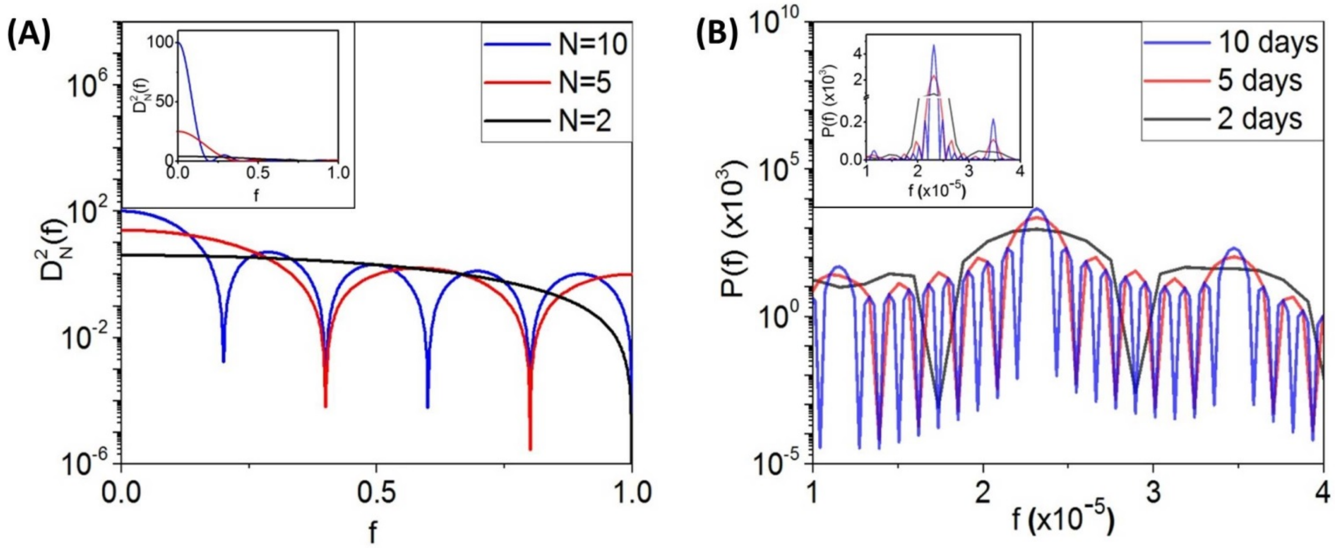

Figure S8. Effect of the length of data on number of peaks for the model and Dirichlet kernel, shown in logarithmic and linear (inset) scales. (A) Square of the Dirichlet kernel plotted for different order N. Order of Dirichlet kernel determines number and position of side peaks. (B) Power spectra of model function $F(t)$ of various lengths. Number and positions of small peaks change with length of simulation, N.

Low pass Butterworth filter

In several previous publications [6–9] a second order digital low pass Butterworth filter was used to remove the high frequency noise typical in locomotor recordings. We show here that, in addition to removing the high frequency noise, filtering can significantly lower the variance of a time series, and in turn, introduce artifactual increase in the apparent statistical significance of features in the filtered power spectrum. We suggest that oversight of this issue may have led to incorrect conclusions in the previous publications.

Assume a filtered time series y_n is obtained from the measured time series x_n as:

$$y_n = H(z)\, x_n \ , \tag{14}$$

where $H(z)$ is a transfer function, given by the expression:

$$H(z) = \frac{b_1 + b_2 z^{-1} + b_3 z^{-2}}{1 + a_2 z^{-1} + a_3 z^{-2}} \tag{15}$$

Here b's and a's are coefficients that are determined from the cutoff period, T_c, and the Nyquist frequency, f_N ($f_N = 1/2t_s$, where t_s is the sampling rate). The filter produces frequency response in the power spectra:

$$G^2(T) = \left| H(e^{-i\frac{2\pi}{T}}) \right|^2 = \frac{G_0^2}{1 + \left(\frac{\tan\left(\frac{\pi}{T}\right)}{\tan\left(\frac{\pi}{T_c}\right)} \right)^{2l}} \ , \tag{16}$$

where G_0 is the response on zero frequency, typically equal to 1, and l is the order of the filter [10]. The frequency response function affects the power spectrum in two obvious ways: it increases the slope for low T values and decreases the total area or total power of the power spectrum.

The relationship between the data variance σ^2 and total power W can be obtained for a classic periodogram [11]. Assume we have a discrete time series x_n with $\bar{x} = 0$ and its discrete Fourier transform is X_n, both of length N. The Fourier transform is determined on a grid of equally spaced frequencies $f_n = n\,\Delta f$, where $\Delta f = f_N/N$. The power spectral density $P(T_n)$, where $T_n = 1/f_n$, can be calculated from Fourier transform as $P(T_n) = |X_n|^2/N$. Then, according to Parseval's theorem

$$\sum_{n=0}^{N-1} x_n^2 = \frac{1}{N}\sum_{n=0}^{N-1} |X_n|^2 = \sum_{n=0}^{N-1} P(T_n)\ , \tag{17}$$

Dividing both sides of the equation by N and using $\sigma^2 = \sum_{n=0}^{N-1} x_n^2/N$ for $\bar{x} = 0$ the above equation becomes

$$\sigma^2 = \frac{1}{N}\sum_{n=0}^{N-1} P(T_n) \tag{18}$$

By multiplying and dividing the right hand side by f_N and using the definition for Δf we have

$$\sigma^2 = \frac{1}{f_N}\Delta f \sum_{n=0}^{N-1} P(T_n) = \frac{1}{f_N}\sum_{n=0}^{N-1} \Delta f\ P(T_n) = \frac{1}{f_N} W = 2 t_s W, \tag{19}$$

where W is the total power. Although filtering does not change t_s, it affects total power W

$$W_{filtered} = \sum_{n=0}^{N-1} \Delta f\, P(T_n)\, G^2(T_n) < \sum_{n=0}^{N-1} \Delta f\, P(T_n) = W_{unfiltered}, \tag{20}$$

Since $\sigma^2 \propto W$, decrease of the total power leads to decrease in data variance, that is

$$\sigma_{filtered}^2 < \sigma_{unfiltered}^2 \tag{21}$$

The Lomb-Scargle method traditionally outputs power spectrum normalized by variance, as this conveniently allows calculation of the significance metric that is independent of σ^2. Since filtering by low pass Butterworth filter does not significantly change $P(T)$ for $T \gg T_c$, but decreases the data variance, the normalized power spectrum $P(T)/\sigma^2$ has higher peaks after filtering.

To further quantify this behavior we introduce a variable R that is equal to the ratio of the height H of a peak in the filtered data to the corresponding peak height h in the unfiltered data (Fig. S8 A). R is inversely proportional to the respective variances

$$R = \frac{H}{h} \propto \left(\frac{\sigma_{filtered}^2}{\sigma_{unfiltered}^2}\right)^{-1} \tag{22}$$

In order to illustrate specific effects of Butterworth filters, we next use it with white noise. For Gaussian white noise, we know that $P(T_n) = \sigma_{unfiltered}^2$. Therefore, the variance after filtering can be expressed as

$$\sigma_{filtered}^2 = 2t_s \sum_{n=0}^{N-1} \Delta f\, P(T_n) G^2(T_n) = 2t_s \sigma_{unfiltered}^2 \sum_{n=0}^{N-1} \Delta f\, G^2(T_n) \tag{23}$$

Combining equations (22) and (23) we find

$$R \propto \frac{\sigma_{unfiltered}^2}{\sigma_{filtered}^2} = \frac{1}{2t_s \sum_{n=0}^{N-1} \Delta f \; G^2(T_n)} \; , \tag{24}$$

indicating R increases linearly with $1/t_s$. Moreover, the summation in the above equation gives area of the frequency response function which decreases with increasing T_c, implying that R should also increase with the cutoff period T_c. In our white noise simulation we used time series generated by averaging 1000 signals with the length of average locomotion recording ($N = 14000$) each produced with the white Gaussian noise Matlab function, 'wgn.m'. We used $l = 2$ low pass Butterworth filter designed with the Matlab function butter.m. Since the filter produces phase shift in data, we used it twice, once in forward direction and once in reverse direction [8].

As predicted by $G(T)$, power spectrum of the filtered noise has slope $\propto T^{2l}$ for low values of T. Additionally, filtering increases overall significance (height) of peaks in LS power spectra (Fig. S9 A). It is seen that while unfiltered time series produces power spectrum without significant periodicities, the same time series after filtering shows multiple 'significant' peaks. As predicted, R changes linearly with $1/t_s$ (Fig. S9 B), and increases with T_c (Fig. S9 C). The ratio R was calculated for the highest peak in the $T > T_c$ regime. For other peaks in this regime the ratio is approximately the same.

The above arguments are applicable to normalized power spectra. If, instead, a power spectrum is not normalized by σ^2, value of the statistical metric (Fig. S9, dashed horizontal lines) decreases due to decrease in σ^2 after filtering, once again resulting in an artifactual increase in the 'significance' of peaks.

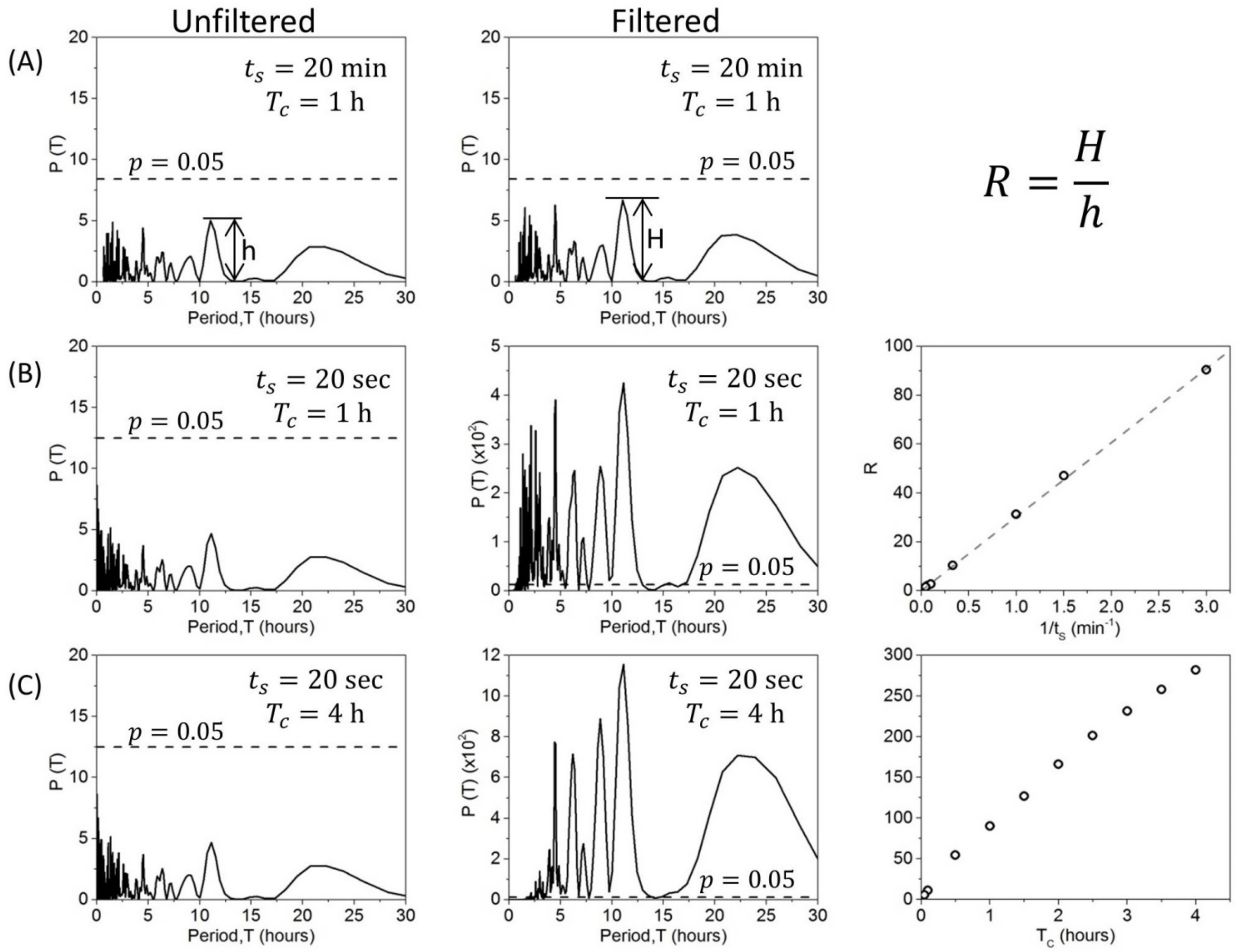

Figure S9. Low pass Butterworth filter increases peak heights in the power spectra of white noise. Power spectrum of unfiltered signal shown on the left; power spectrum of filtered signal shown in the middle. (A) To quantify and analyze peak increase we introduce variable R. Small peak increase shown in signal with 20 min bin size (t_s) and cutoff period (T_c) 1 h. (B) R proportional to $1/t_s$. Example power spectra shown for $t_s = 20$ sec and $T_c = 1$ h. Peak height ratio is larger than that for $t_s = 20$ min shown in (A). (C) R increases with increase of T_c. Power spectra shown for $t_s = 20$ sec and $T_c = 4$ h. Peak height ratio is significantly larger than that for $T_c = 1$ h shown in (B).

In order to see whether these effects in power spectra depend on the method of calculating $P(T)$, we repeated the analysis with MESA. Similar to the case with Lomb-Scargle, we find that filtering introduces artificial increase in peak heights for $T \gg T_c$ in MESA calculations.

The effect of filtering on peak significance can be observed in all types of signal, but it is more pronounced in noisy data in which power is spread equally in the frequency domain. Therefore, this effect is much stronger in the analysis of arrhythmic animals, which have activity most similar to noisy time series. We therefore do not recommend usage of digital filters, especially when low-power features in power spectrum are of interest.

Statistical distribution of circadian periods

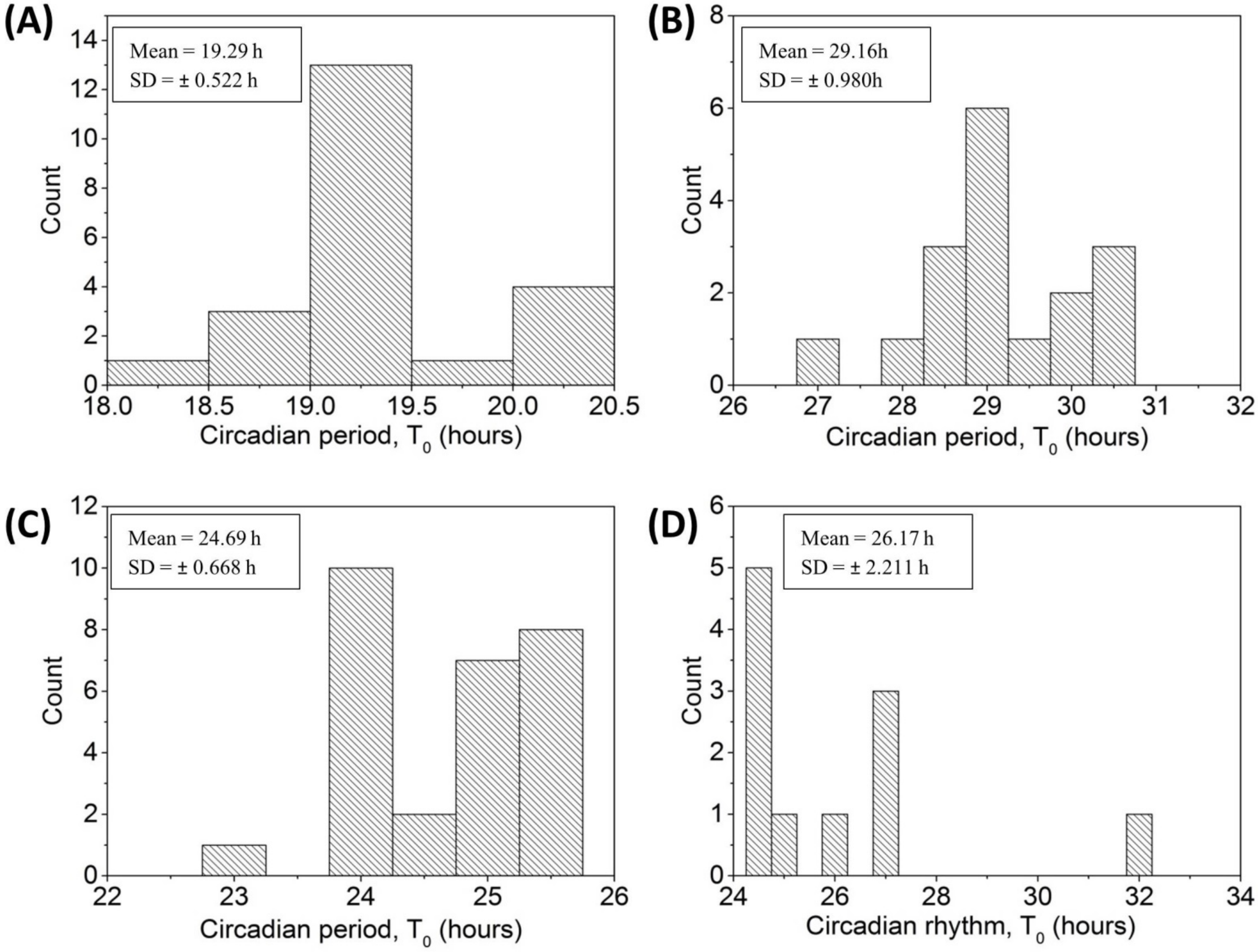

Figure S10. Circadian period distribution for measured per^S (A), per^L (B), wild type (C) and tim^{UL} (D) flies in DD. Mean and standard deviation for each genotype is given in insets.

References

1. Allada R, Kadener S, Nandakumar N, Rosbash M: **A recessive mutant of *Drosophila* Clock reveals a role in circadian rhythm amplitude.** *EMBO J* 2003, **22**:3367–3375.

2. Allada R, White NE, So W V, Hall JC, Rosbash M: **A mutant *Drosophila* homolog of mammalian Clock disrupts circadian rhythms and transcription of period and timeless.** *Cell* 1998, **93**:791–804.

3. Baylies MK, Bargiello TA, Jackson FR, Young MW: **Changes in abundance or structure of the per gene product can alter periodicity of the *Drosophila* clock.** *Nature* 1987, **326**:390–392.

4. Prendergast BJ, Cisse YM, Cable EJ, Zucker I: **Dissociation of ultradian and circadian phenotypes in female and male Siberian hamsters.** *J Biol Rhythms* 2012, **27**:287–298.

5. Harris FJ: **On the use of windows for harmonic analysis with the discrete Fourier transform**. *Proc IEEE* 1978, **66**:51–83.

6. Power JM, Ringo JM, Dowse H: **The effects of period mutations and light on the activity rhythms of *Drosophila melanogaster***. *J Biol Rhythms* 1995, **10**:267–280.

7. Dowse H: **Maximum entropy spectral analysis for circadian rhythms: theory, history and practice**. *J Circadian Rhythms* 2013, **11**:6.

8. Levine JD, Funes P, Dowse H, Hall JC: **Signal analysis of behavioral and molecular cycles.** *BMC Neurosci* 2002, **3**:1.

9. Blum ID, Zhu L, Moquin L, Kokoeva M V, Gratton A, Giros B, Storch K-F: **A highly tunable dopaminergic oscillator generates ultradian rhythms of behavioral arousal.** *Elife* 2014, **3**:e05105.

10. Shenoi BA: *Introduction to Digital Signal Processing and Filter Design*. Hoboken, New Jersey.: John Wiley & Sons, Inc.; 2006.

11. Percival DB, Walden AT: *Spectral Analysis for Physical Applications*. Cambridge University Press; 1993.
